# Novel *Lom-dh* Genes Play Potential Role in Promoting Egg Diapause of *Locusta migratoria* L.

**DOI:** 10.3389/fphys.2019.00767

**Published:** 2019-06-18

**Authors:** Kun Hao, Xiongbing Tu, Hidayat Ullah, Mark Richard McNeill, Zehua Zhang

**Affiliations:** ^1^State Key Laboratory for Biology of Plant Diseases and Insect Pests, Institute of Plant Protection, Chinese Academy of Agricultural Sciences, Beijing, China; ^2^Department of Agriculture, University of Swabi, Khyber Pakhtunkhwa, Pakistan; ^3^Canterbury Agriculture and Science Centre, AgResearch, Lincoln, New Zealand

**Keywords:** diapause hormone, diapause induction, tandem repeat, RNAi, neuropeptide

## Abstract

Diapause hormone (DH) neuropeptides in insects are produced by the genes belonging to *pban/capa* family. Previous studies show that DH contains a conserved sequence of WFGPRXa that plays vital role in diapause regulation of some Lepidopteran species. However, the function of DH in other species is still unknown. In order to expand our understanding of DH function in diapause induction, *Lom-pban*, *Lom-capa*, and five candidates DH precursor genes (*Lom-dh1*, *Lom-dh2*, *Lom-dh3*, *Lom-dh4*, *Lom-dh5*) of *Locusta migratoria* L. were subsequently cloned. We identified *Lom-dh1* to *Lom-dh5* as novel genes that encoded five types (type I–V) of 44 tandem repeats of DH-like neuropeptides, which might promote egg diapause of *L. migratoria*. To test this hypothesis, we identified four types of eight new neuropeptides encoded by *Lom-dh* using liquid chromatography–tandem mass spectrometry from the central neuron system of *L. migratoria* under both short (10:14 L:D) and long (16:8 L:D) photoperiods. Later on, we synthesized four type I DH-like neuropeptides, LDH1, SDH1, LDH2, and SDH2, encoded by *Lom-dh2*/*Lom-dh3* and injected them into fifth instar female locusts. Egg diapause incidences were observed after female oviposition. The four DH-like neuropeptides significantly increased the incidence of egg diapause under the short photoperiod, but the response was absent under the long photoperiod. Injection of ds*Lom-dh* into female adults of *L. migratoria* under the short photoperiod could inhibit egg diapause, with no response under the long photoperiod. This study identified a new member of *pban/capa* family being the second example beside *Bombyx mori*, where the DH showed significant role on maternal induction of diapause.

## Introduction

The PRXamide family of peptides plays essential roles in insect metabolism, including mating, development, diapause, muscle contraction, and induction of cuticular melanization (Jurenka and Nusawardani, 2011). Leucopyrokinin, the first PRXamide family member, was identified in the cockroach *Leucophaea maderae* (Holman et al., 1986). Since then, other members of PRXamide family, including CAPA peptides, pyrokinins (PK), ecdysis-triggering hormone, periviscerokinins (PVKs), tryptopyrokinins (TPKs), diapause hormones (DHs), and pheromone biosynthesis-activating neuropeptides (PBANs), have also been identified (Holman et al., 1986; Veenstra, 2014; Jurenka, 2015). Usually, *pban* family genes are firstly translated into a large inactive protein precursor, and then cleaved by endonuclease and amidation to produce active neuropeptides (Camargo et al., 2012). CAPA and PBAN are two of the most important multifunctional proteins and their gene sequences and structures have been thoroughly studied to reveal their roles in insect development and diapause regulation (Nagasawa et al., 1994; Loi and Tublitz, 2004; Uehara et al., 2011). Most CAPA/PBAN families share the common sequence “FXPRLamide.” The last amino acid of “FXPRLamide” is variable, including L, N, V, and I (Coast and Schooley, 2011; Veenstra, 2014; Jurenka, 2015). Insect CAPA/PBAN peptides are encoded by *capa* and *pban* gene. There are three PK peptides (PK-1, PK-2, PK-3), one DH-like peptide (DH-2), and one PBAN peptide (PBAN), expressed by the *pban* gene in almost all insects (Jurenka and Nusawardani, 2011). The DH-2 peptide is usually characterized by a GMWFGPRLamide ending, with two CAPA peptides (CAPA-1, CAPA-2) and one DH-like peptide (DH-1) expressed by the capa gene. The DH-1 sequence found in the *capa* gene of insects is also highly conserved with a consensus sequence of GMWFGPRLamide (Jurenka, 2015). Notable exceptions include *Anopheles gambiae* and *L. migratoria* for which the consensus sequences are AMWFGPRLamide and PLWFGPRVamide, respectively (Predel and Wegener, 2006). Hence, DH-1 and DH-2 are mostly encoded by a consensus sequence of GMWFGPRLamide. The DH-1 (from CAPA) and DH-2 (from PBAN) of the red flour beetle (*Tribolium castaneum*) have the same consensus sequence, but the strength of binding with its receptors is totally different. The binding affinities between DHs and DH receptors indicated that DH-1 is more selective for its respective authentic receptors than DH-2 (Jiang et al., 2014). This difference in binding affinity may cause DH-1 and DH-2 species to perform variably (Altstein et al., 2013). There has been functional divergence in DH-1 and DH-2 with DH-2 from PBAN linked to diapause regulation in Lepidoptera, but there is no evidence indicating that DH-1 from CAPA has a similar role (Xu et al., 1995; Xu and Denlinger, 2004; Predel and Neupert, 2007; Schmitt et al., 2015). Evidence indicates that the function of DH-1 and DH-2 have diverged in the PRXamide family (Jiang et al., 2014).

Diapause hormone can inhibit synthesis of cGMP, where the reduction of cGMP leads to increased trehalase activity of ovaries to enhance or stimulate the incorporation of glucose into oocytes through the catabolism of hemolymph trehalose in *B. mori* (Yamashita, 1981; Ikeda et al., 1993). However, how the environmental signals induced insect diapause by DH is still unknown. *L. migratoria* is an important insect pest in many parts of Africa, Asia, and Australia (Turkez et al., 2014) with facultative embryonic egg diapause. Unlike most insects, diapause induction of locusts is a trans-generation process, but similar to silkworm *B. mori*. Changes due to environment in the maternal parent transfer diapause factor to the offspring eggs (Hakomori and Tanaka, 1992; Tanaka, 1992; Tanaka, 1994). The offspring eggs of locusts need to be induced under a low temperature until it ceased development in late anatrepsis stage before embryo entered the diapause (Wardhaugh, 2010). Our previous work has established the conditions for diapause induction in *L. migratoria*. A temperature ranging from 27 to 30°C can induce diapause in *L. migratoria* under short photoperiod. Our previous study also demonstrated that insect hormone biosynthesis, the insulin signaling pathway and the peroxisome proliferator-activated receptor (PPAR) signaling pathway were involved in diapause regulation of the locust eggs (Tu et al., 2015; Hao et al., 2017). Meanwhile, the low level of ecdysteroids in both maternal and offspring eggs was observed in the diapause process, whereas the high quantity of ecdysteroids terminated the diapause in *L. migratoria* (Tawfik et al., 2002). Previous studies on *L. migratoria* have identified 14 neuropeptides (pyrokinin), two genes in the CAPA/PBAN family, and predicted four TPKs (Clynen et al., 2003; Coast and Schooley, 2011; Veenstra, 2014; Hou et al., 2015). The C-terminal consensus sequences in parts of these neuropeptides and genes composed of WFGPRXa (X = V, I, or Y) are similar to those in Lepidoptera DH-2 (Altstein et al., 2013; Zhang et al., 2015). The locust pyrokinin neuropeptides elicit significant diapause-inducing activity in the lepidopteran silkworm (Nachman et al., 1993). We believe that some of these DH-like neuropeptides may be involved in induction of *L. migratoria* embryonic diapause (Tanaka and Zhu, 2008; Tu et al., 2015). The functional genes or neuropeptides belonging to CAPA or PBAN in locusts have not yet been studied. Even it is unknown whether gene function divergence is occurring in locusts? Hence, in this study, we cloned the entire *pban/capa* family genes of *L. migratoria*. We identified novel *Lom-dh* genes and DH-like neuropeptides, and even applied injection bioassay and RNAi assay to understand their functions in insect diapause regulation.

## Results

### Prediction and Cloning of Pyrokinin Family Genes in *L. migratoria*

To obtain the *L. migratoria capa/pban* gene sequences, we first aligned other insect taxa CAPA/PBAN family protein precursors with the *L. migratoria* genome and found 28 target contigs (Supplementary Table S1). Among these, only five contigs, including AVCP010882506 (TPK1), AVCP010410303 (TPK2), AVCP010159529 (TPK3), AVCP010159522 (TPK4), and AVCP010410310 (TPK5), contained the conserved amino acid sequence FGPRV/I/Y of CAPA/PBAN, whereas the other 22 contigs were non-target sequences (Supplementary Table S1). We then aligned other insect taxa CAPA/PBAN family protein precursors once again with the *L. migratoria* transcriptome (accession number: GCGJ00000000) in NCBI, and found two new target transcripts, including gb| GCGJ01004763.1| (*Lom-capa*) and gb| GCGJ01044456.1| (*Lom-pban*) (Supplementary Table S1). We aligned these two new transcripts with the *L. migratoria* genome, and six other contigs, including the *capa* gene of gb| AVCP011143372.1|, gb| AVCP011143377.1|, gb| AVCP011143364.1|, gb| AVCP011143367.1|, and the *pban* gene of gb| AVCP011098992.1|, gb| AVCP011098990.1| (Supplementary Table S1). Finally, we aligned 14 pyrokinin family peptides with the *L. migratoria* genome and found a contig of gb| AVCP010267064.1| (Lom-PK-2) in NCBI (Supplementary Table S1). In total, 12 contigs related to CAPA/PBAN in *L. migratoria* genome were obtained. Among them, genome sequences of TPK 1, 3, 4, 5, and mRNA sequence of *Lom-capa*, *Lom-pban* had complete coding sequences (CDS), TPK 2 lacked the 5′-end, whereas *Lom-PK-2* lacked both a 5′- and 3′-end (Supplementary Figure S1). CDS analysis indicated TPK4 and TPK5 had high identity, in the similar manner as that of TPK2 and TPK3.

Then, *Lom-TPK1* (789 bp), *Lom-TPK2* (1512 bp), *Lom-TPK3* (1767 bp), *Lom-TPK4* (732 bp), and *Lom-TPK5* (1092 bp) were cloned from the *L. migratoria* genome DNA and central neural system cDNA, respectively. *Lom-pban* (498 bp, GenBank accession number: MG517529) and *Lom-capa* (846 bp, GenBank accession number: MG517530) were cloned from central neural system cDNA of *L. migratoria.*

### Phylogenetic and Codon Bias Analysis of *L. migratoria* Pyrokinin Family Genes

To compare the phylogenetic position of these neuropeptide precursors, we downloaded all insect CAPA/PBAN precursors into NCBI and together with Lom-CAPA, Lom-PBAN, Lom-TPK1, Lom-TPK2, Lom-TPK3, Lom-TPK4, and Lom-TPK5 were used to construct phylogenetic trees. Previous research showed that the length of CAPA/PBAN family precursors differed. Within the same insect order, they were conserved in similar positions (Supplementary Figure S2), whereas in different orders, they had only the consensus conserved sequence of FXPRL/V/I. Thus, the clustering results were unable to illustrate the phylogenetic position of all insects CAPA/PBAN. The pyrokinin precursors of Hymenoptera, Lepidoptera, Diptera, and *L. migratoria*, however, were well clustered into CAPA and PBAN (Supplementary Figure S2). Thus, *L. migratoria* contains both CAPA and PBAN. Lom-TPK1, 2, 3, 4, and 5 were clustered into CAPA (Supplementary Figure S2). However, Lom-TPK1, 2, 3, 4, and 5 are clustered together on a unified branch, and are not clustered with Lom-CAPA.

Codon bias analysis showed that the CAI of *Lom-pban* and *Lom-capa* are 0.02–0.03; the CBI of *Lom-pban* and *Lom-capa* are about 0.5. The FOP of *Lom-pban* and *Lom-capa* are about 0.7 and GC3s of *Lom-pban* and *Lom-capa* are about 0.9. CAI of *Lom-TPK1, 2, 3, 4*, and *5* are 0.06–0.09; CBI of *Lom-TPK1, 2, 3, 4*, and *5* are −0.2 – 0.1; FOP of *Lom-TPK1, 2, 3, 4*, and *5* are 0.2–0.4; and GC3s of *Lom-TPK1, 2, 3, 4*, and *5* are about 0.3–0.6. CAI of the reference gene *actin*, *gapdh*, and *ef* are 0.1–0.2; CBI of *actin*, *gapdh*, and *ef* are about 0.0; FOP of *actin*, *gapdh*, and *ef* are about 0.4; and GC3s of *actin*, *gapdh*, and *ef* are 0.4–0.5. CAI, CBI, FOP, and GC3s values of *Lom-pban* and *Lom-capa* are significantly different from reference genes and *Lom-TPKs*. However, there is no significant difference between CBI, FOP, and GC3s values of *Lom-TPKs* and the reference genes (Supplementary Figure S3). Thus, we considered that *Lom-TPK1, 2, 3, 4*, and *5* are novel types of genes from *Lom-capa* and *Lom-pban* and then we renamed *Lom-TPK1*, *2*, *3*, *4*, and *5* as *Lom-dh1*, *2*, *3*, *4*, and *5* (GenBank accession number: MG517531, MG517532, MG517533, MG517534, MG517535), respectively.

### Lom-CAPA/PBAN/DH Structure Analysis

**FIGURE 1:**
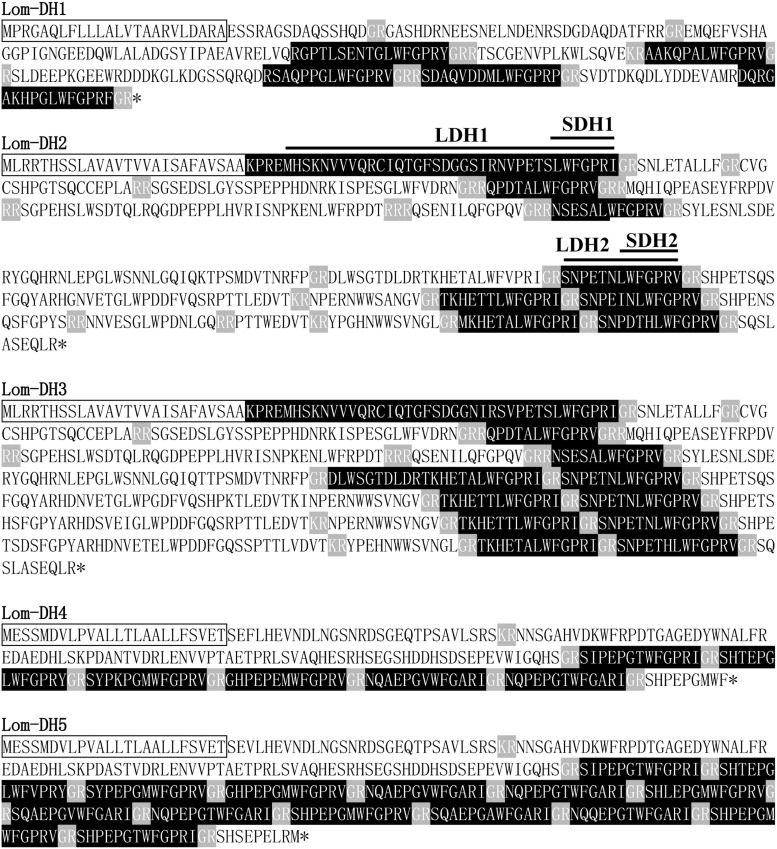
Lom-DH1 to Lom-DH5 protein sequences. Amino acid sequences in black box indicate signal peptide, gray amino acid sequences indicate endoproteolytic cleavage sites, and black amino acid sequences indicate short neuropeptides.

**FIGURE 2:**
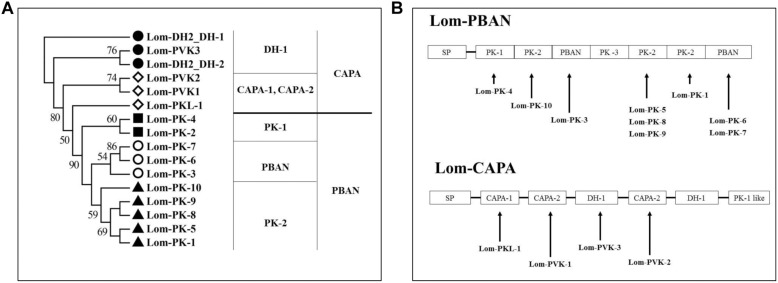
Neuropeptides encoded by *capa/phan* in *Locusta migratoria*. **(A)** Phylogenetic analysis of pyrokinin consensus sequences. **(B)** Genes of coding neuropeptide; solid line indicates the location of the neuropeptides in the *capa/pban* genes.

We used ORF Predictor to identify the signal peptide and endoproteolytic cleavage site of CAPA/PBAN neuropeptide precursors. This indicated that the new cloned *Lom-dh1* to *Lom-dh5* genes had no intron or any other alternative splicing transcripts (Figure 1). Furthermore, potential signal peptides of Lom-DH1, 2, 3, 4, and 5 were predicted and demonstrated in Figure 1. All potential neuropeptides could be processed at possible endoproteolytic cleavage sites *GR*, *GRR*, *KR* (Figure 1). Gene structure analysis showed that Lom-DH2 contained eight DH-like neuropeptides, whereas Lom-DH3 contained 11 DH-like neuropeptides. Lom-DH1 contained five DH-like neuropeptides, Lom-DH5 contained 14 DH-like neuropeptides, and Lom-DH4 contained six DH-like neuropeptides (Figure 1). According to the C-terminus consensus sequences, 44 DH-like neuropeptides, encoding by Lom-DH1 to Lom-DH5, can be classified into five types (Table 1). We used Weblogo to build a sequence logo based on consensus sequences of the 13 C-terminal amino acids of all 44 DH-like neuropeptides. The results indicated that two of the consensus amino acid sequences, including LWFGPRI and LWFGPRV appeared most frequently (Supplementary Figure S4). These two amino acid sequences were clustered on the reported *L. migratoria* neuropeptides. Results indicated they belonged to DH-1 part of the *capa* gene, the same as Lom-PVK-3 (Figure 2A).

**TABLE 1:** DH-like neuropeptides classified into five types by C-terminus consensus sequences.

**C-terminus consensus sequences**	**Type**	**Amount**
LWFGPRXa (X = V, I, Y, P, F)	I	27
MWFGPRVa	II	7
TWFGPRIa	III	3
TWFGARIa	IV	4
VWFGARIa	V	3
Total	5 types	44

We aligned the *Lom-capa* and *Lom-pban* genes to the neuropeptides previously found in *L. migratoria* and found that 14 neuropeptides, except for Lom-PK-2, were encoded by the *Lom-capa* or *Lom-pban* genes (Table 2). The gene structure of *Lom-capa* was SP_CAPA-1_CAPA-2_DH-1_CAPA-2_DH-1_PK-1 with the segments of CAPA-2_DH-1 repeated two times (Figure 2B). Although the gene structure of *Lom-pban* was SP_PK-1_PK-2_PBAN_PK-3_PK-2_PK-2_PBAN, there was no DH, with PK-2_PBAN segments repeated twice (Figure 2B). The amino acid sequence of PK-3 was DPPVDGPLVWLPLQVSPRL in the *pban* gene, but there was no neuropeptide corresponding to PK-3 indicated for *L. migratoria*. The absence of a PK-3 neuropeptide, even though sequences were present in the *pban* gene, is potentially caused by a mutation from GR to AR at the endoproteolytic cleavage site, which prevents peptide expression. Thus, the *Lom-capa* and *Lom-pban* genes are unique. The former could be defined as the traditional *capa* gene, and the latter could be defined as the traditional *pban* gene when compared with other insect taxa. Most importantly, the *capa* gene, including DH-1, may be involved in locust diapause regulation, whereas the *pban* gene plays no role in this process.

**TABLE 2:** Pyrokinin family neuropeptides identified in *Locusta migratoria* L.

**Neuropeptide**	**Amino acid sequence**	**M (Da)**
**Pyrokinin family neuropeptides of *L. migratoria* identified in a previous study** (Clynen and Schoofs, 2009)		
Lom-PK-1	DSGDEWPQQPFVPRLa	1768.9
Lom-PK-2	pQSVPTFTPRLa	1126.6
Lom-PK-3	GAVPAAQFSPRLa	1211.7
Lom-PK-4	EGDFTPRLa	932.5
Lom-PK-5	RQQPFVPRLa	1138.7
Lom-PK-6	RLHQNGMPFSPRLa	1550.8
Lom-PK-7	X_1_HX_2_NGMPFSPRX_1_a	1394.7
Lom-PK-8	pQX_2_PFVPRX_1_a	965.5
Lom-PK-9	X_2_PFVPRX_1_a	854.5
Lom-PK-10	VX_1_AGPFVPRX_1_a	1066.7
Lom-PKL-1	TSSLFPHPRLa	1152.6
Lom-PVK-1	AAGLFQFPRVa	1103.6
Lom-PVK-2	GLLAFPRVa	870.5
Lom-PVK-3	DGGEPAAPLWFGPRVa	1566.8
**Pyrokinin family neuropeptides identified by LC-MS/MS in this study**		
Lom-PK-1	DSGDEWPQQPFVPRLa	1768.8533
Lom-PK-3	GAVPAAQFSPRLa	1211.6775
Lom-PK-4	EGDFTPRLa	932.4716
Lom-PK-5	RQQPFVPRLa	1138.6724
Lom-PK-8	pQQPFVPRLa	965.5447
Lom-PK-10	**ESAEQGGGVSAWQGGEPQQEE QVLAGPFVPRLa**	3335.6169
Lom-PKL-1	**pQDGDKGISKLKKTSSLFPHPRIa**	2433.3491
Lom-PVK-1	AAGLFQFPRVa	1103.6239
Lom-PVK-2	**RGLLAFPRVa**	1026.645
Lom-PVK-3	**DGGQPAAPLWFGPRVa**	1565.8102
**Lom-PK-11**	**KGLVASARVa**	898.5712
**Lom-DH-1 (type I)**	**SDAQVDDMLWFGPRPa**	1731.8038
**Lom-DH-2 (type I)**	**AQPPGLWFGPRVa**	1322.7247
**Lom-DH-3 (type I)**	**AAKQPALWFGPRVa**	1438.8197
**Lom-DH-4 (type I)**	**GAKHPGLWFGPRFa**	1467.7887
**Lom-DH-5 (type II)**	**SHPEPGMWFGPRVa**	1494.719
**Lom-DH-6 (type II)**	**HPEPGMWFGPRVa**	1407.687
**Lom-DH-7 (type IV)**	**NQPEPGTWFGARIa**	1470.7368
**Lom-DH-8 (type V)**	**SQAEPGVWFGARIa**	1415.731

*Mono-isotopic masses (M) are given in dalton (Da); disulphide bridges are taken into account (one S–S = M – 2 Da); pQ, pyroglutamic acid; a, amide; X_1_, leucine or isoleucine; X_2_, lysine or glutamine. Novel peptides are marked in bold.*

### Novel Pyrokinin Family Neuropeptides Identified by LC-MS/MS

These results demonstrate that most of the 14 PK neuropeptides obtained by previous MS are encoded by *Lom-pban* and *Lom-capa* (Figure 2B). We performed LC-MS/MS to identify the neuropeptides from the central neuron system of *L. migratoria* under short and long photoperiods. We identified all of the 14 PK neuropeptides in previous studies, except Lom-PK-2, Lom-PK-6, and Lom-PK-7. There were minute differences between the 14 PK and our newly identified peptides. The N-terminal of the newly identified Lom-PK-10, Lom-PKL-1, and Lom-PVK-2 is longer than the previously identified ones (Table 2). The terminal amino acid of Lom-PKL is I rather than L (Table 2). Similarly, the fourth amino acid of Lom-PVK-2 is Q rather than E (Table 2). In addition, we identified nine new neuropeptides encoded by *Lom-dh1* to *Lom-dh5* genes and named them Lom-PK-11, Lom-DH-1, Lom-DH-2, Lom-DH-3, Lom-DH-4, Lom-DH-5, Lom-DH-6, Lom-DH-7, and Lom-DH-8. Lom-DH-1, Lom-DH-2, Lom-DH-3, and Lom-DH-4 were type I DH-like neuropeptides. Lom-DH-5 and Lom-DH-6 were type II DH-like neuropeptides. Lom-DH-7 was a type IV DH-like neuropeptide. Lom-DH-8 was a type V DH-like neuropeptide (Table 2).

### DH Function Identified by Injection Assay

**FIGURE 3:**
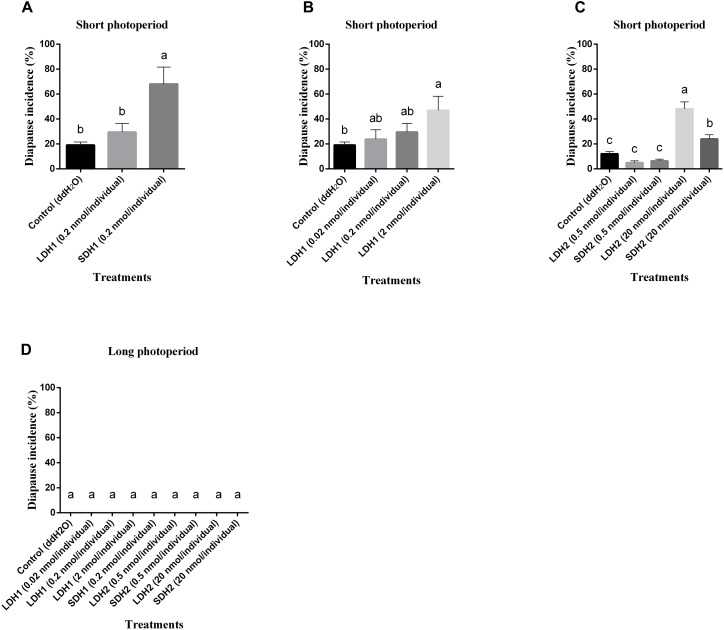
Diapause incidence of *L. migratoria* eggs after treatment of female parent with selected doses of LDH1 (MHSKNVVVQRCIQTGFSDGGSIRNVPETSLWF GPRIa), SDH1 (LWFGPRIa), LDH2 (SNPETNLWFGPRVa) and SDH2 (LWFGPRVa). **(A)** Injection of 0.2 nmol/individual of LDH1 or SDH1 at 10:14 L:D and 28°C. **(B)** Injection of 0.02, 0.2, and 2 nmol/individual LDH1 under 10:14 L:D and 28°C. **(C)** Injection of 0.5 and 20 nmol/individual of LDH2 or SDH2 under 10:14 L:D and 28°C. **(D)** Injection of 0.02, 0.2, and 2 nmol/individual LDH1, 0.2 nmol/individual of SDH1, 0.5, and 20 nmol/individual of LDH2 or SDH2 at 16:8 L:D and 28°C. Control treatment was injection of distilled water (control). The lowercase a, b, and c indicated that statistically significant difference of diapause incidence was considered on an error probability of *p* < 0.05 by one-way ANOVA using Duncan’s multiple-range test.

To confirm locust DH function, LDH1 (MHSKNVVVQRC IQTGFSDGGSIRNVPETSLWFGPRIa), SDH1 (LWFGPRIa), LDH2 (SNPETNLWFGPRVa) and SDH2 (LWFGPRVa) as shown in Figure 1 were synthesized and injected into fifth instar females independently. All of the treated females turned to adults’ within 72 h of neuropeptides injection. After attaining the adulthood, the insects were reared for additional 14 days afterward they reached to maturity and began eggs laying. The control treatment consisted of fifth instar females injected with distilled water. Under an L:D 10:14 photoperiod and constant temperature of 28°C, we induced diapause in 6.8–24.7% of the eggs oviposited by females treated with LDH1, SDH1, LDH2, and SDH2. This allowed us to test whether DH enhanced or inhibited diapause. Results showed that SDH1 induced diapause in eggs under L:D 10:14 at a constant temperature of 28°C, with 68.1% of the eggs entering diapause after injection of SDH1 at 0.2 nmol/individual. This was significantly higher than the diapause incidence after injection of LDH1 at 0.2 nmol/individual and control (*F* = 9.344, *P* = 0.001) (Figure 3A). In the control, 19.1% (range from 10.1 to 24.7%) of the eggs entered diapause (Figures 3A,B). However, no eggs were induced to enter diapause by injection of LDH1, SDH1, LDH2, and SDH2 among the eggs laid and incubated under long photoperiod at 28°C, even though 2 nmol/individual was injected (Figure 3D).

There was a dose-related effect on diapause induction. Injection of LDH1 at 2 nmol/individual produced 47.0% diapause eggs compared with 23.8% and 30.0% when the dose was 0.02 and 0.2 nmol/individual, respectively (*F* = 2.407, *P* = 0.105) (Figure 3B). Under short photoperiod (L:D 10:14) and at a constant temperature of 28°C, injection of 20 nmol/individual of both LDH2 and SDH2 significantly induced diapause (*F* = 30.197, *P* = 0.001), but at a lower dose of 0.5 nmol/individual, there was no significant difference between the LDH2, SDH2 treatments and the control group (Figure 3C). No eggs entered diapause when LDH2 and SDH2 were injected into females held under long photoperiod (L:D 16:8) and at a constant temperature of 28°C (Figure 3D).

SDH1 and LDH1 could promote diapause at 0.2 and 2 nmol/individual, respectively. Meanwhile, the dose of 20 nmol/individual promoted diapause in LDH2 and SDH2 treatments. The results also indicated that these synthetic DH peptides could effectively promote locust diapause at a specific dose rate.

### *Lom-dh* Genes Function Identified by RNAi

**FIGURE 4:**
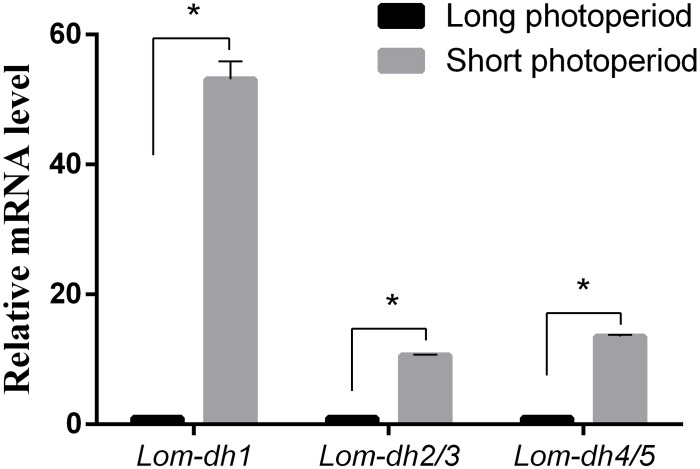
Relative mRNA level of *Lom-dh* genes detect in *L. migratoria* under long and short photoperiod. ^*^Indicates an error probability of *p* < 0.05 by Student’s *t*-test.

**FIGURE 5:**
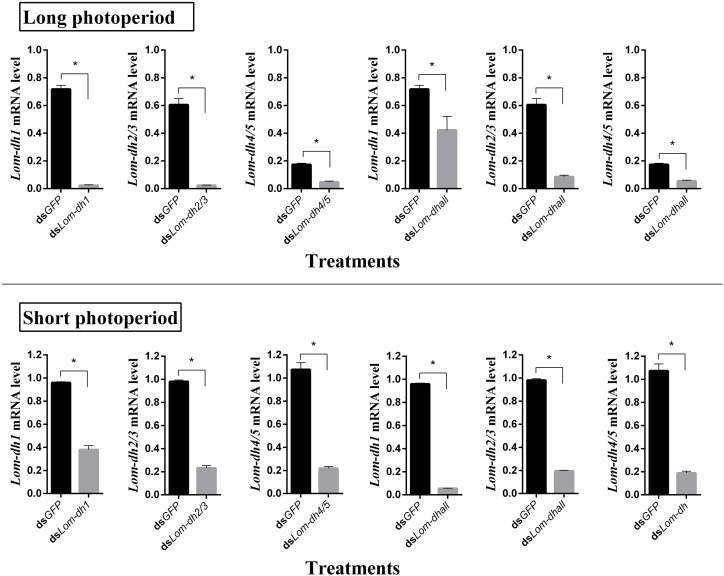
RNAi efficiency of *Lom-dh* genes detect in *L. migratoria* under long and short photoperiod. ^*^Indicates an error probability of *p* < 0.05 by Student’s *t*-test.

**FIGURE 6:**
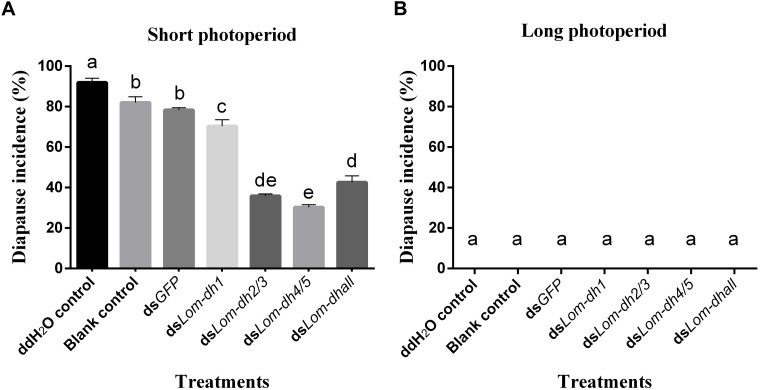
**(A)** Diapause incidence after injecting ds*Lom-dh* to RNAi *Lom-dh* genes of *L. migratoria* under short photoperiod. **(B)** Diapause incidence after injecting ds*Lom-dh* to RNAi *Lom-dh* genes of *L. migratoria* under long photoperiod. The lowercase a, b, c, d, and e indicated that statistically significant difference of diapause incidence was considered on an error probability of *p* < 0.05 by one-way ANOVA using Duncan’s multiple-range test.

Expression levels of *Lom-dh1*, *Lom-dh2/3*, and *Lom-dh4/5* were significantly higher (53.1, 10.6, and 13.5 folds) in maternal locust under short photoperiod as compared to long photoperiod (Figure 4). This demonstrated that the *Lom-dh* genes might have the function of diapause induction. To verify the function of all *Lom-dh* genes, ds*Lom-dh1*, ds*Lom-dh2/3*, ds*Lom-dh4/5*, and ds*Lom-dhall* were injected into adult female *L. migratoria* maintained under L:D10:14 and L:D16:8 photoperiods and at a constant temperature of 28°C. Because *Lom-dh2* and *Lom-dh3* shared a common region, hence the *Lom-dh2* and *Lom-dh3* were knocked down simultaneously by the same dsRNA (ds*Lom-dh2/3*). Similarly, the *Lom-dh4* and *Lom-dh5* were knocked down by the dsRNA (ds*Lom-dh4/5*). ds*Lom-dhall*, which was used to knock down all *Lom-dh* at once, was a mixture of ds*Lom-dh1*, ds*Lom-dh2/3*, and ds*Lom-dh4/5*. Then we performed RT-PCR to check RNAi efficiency. Relative mRNA levels of ds*Lom-dh1*, ds*Lom-dh2/3*, ds*Lom-dh4/5*, and ds*Lom-dhall* treatments were reduced significantly compared with the control and ds*GFP* treatments under both L:D 10:14 and L:D 16:8 photoperiods (Figure 5). Females were maintained under these conditions until they reached maturity and commenced eggs laying. Diapause incidences in the control group and ds*GFP* treatment ranged from 76.23 to 85.59% under L:D 10:14 and at a temperature of 28°C. Meanwhile diapause incidences were 70.34% in ds*Lom-dh1* treatment, 35.84% in ds*Lom-dh2/3* treatment, 30.27% in the ds*Lom-dh4/5* treatment, and 42.59% in the ds*Lom-dhall* treatment at L:D 10:14, 28°C. Compared with the control and the ds*GFP* treatments, egg diapause incidences were reduced significantly in the ds*Lom-dh1*, ds*Lom-dh2/3*, ds*Lom-dh4/5*, and ds*Lom-dhall* treatments (*F* = 117.891, *P* = 0.001) (Figure 6A). No eggs entered diapause resulting from injection of ds*Lom-dh1*, ds*Lom-dh2/3*, and ds*Lom-dh4/5* where the eggs were laid and incubated under long photoperiod at 28°C (Figure 6B). Results revealed that diapause incidences were significantly down regulated after the knock down of *Lom-dh* genes independently or jointly. This indicated that all *Lom-dh* genes could induce diapause of *L. migratoria*.

## Discussion

### Novel DH Neuropeptides Encoded by Novel *Lom-dh* Genes

Most TPK genes of Orthoptera occur in the *capa/pban* gene family (Veenstra, 2014). We cloned the entire *capa/pban* family genes of *L. migratoria* in our study. Similar to other species, *Lom-pban* and *Lom-capa* had introns. Unlike *Lom-pban* and *Lom-capa*, there were no introns in TPKs (*Lom-TPK1, 2, 3, 4*, and *5*). Phylogenetic analysis showed that the TPKs in *L. migratoria* were similar to *capa*, but not identical. Codon bias analysis also identified a significant difference between *capa/pban* and TPKs. Thus, we renamed TPK (*Lom-TPK1, 2, 3, 4*, and *5*) as *Lom-dh* (*Lom-dh1, 2, 3, 4*, and *5*) in *L. migratoria* to better understand their function and avoid classification confusion. *Lom-dh* is a new type of DH precursor gene in addition to *Lom-capa* and *Lom-pban.*

The *pban/capa* family is involved in insect growth and development. Diapause is either induced or inhibited by the same gene as seen in some species of Lepidoptera, but their different segments may play different roles (Hasegawa, 1957; Jurenka, 2015; Zhang et al., 2015). For example, the PBAN segment of the *pban* gene regulates growth and development and the DH-2 segment regulates diapause. In the capa gene, CAPA-1 and the CAPA-2 segment regulate growth and development (Zhang et al., 2015). In *L. migratoria*, however, no DH-2 segment was found in the Lom-PBAN, CAPA-1_CAPA-2_DH-1 segments contained in Lom-CAPA. Moreover, when we compared all neuropeptides of Lom-PBAN, Lom-CAPA, and Lom-DH1-5 with former studies (Clynen and Schoofs, 2009), we found the former Lom-PVK3 (DH-1 from Lom-CAPA) and four types of eight novel DH neuropeptides (Lom-DH-1 to Lom-DH-8) encoded by the five novel *Lom-dh* genes.

Most reported CAPA or PBAN precursors in Diptera, Hymenoptera, and Lepidoptera also contained the DH segment (Jurenka, 2015; Zhang et al., 2015). *L. migratoria* PBAN had no DH segment, similar to *Rhodnius prolixus* Stål (Hemiptera: Reduviidae), *Lygus hesperus* Knight (Hemiptera: Miridae), and *Drosophila melanogaster* Meigen (Diptera: Drosophilidae) (Supplementary Table S2), but while CAPA was found to include a DH segment, it was dissimilar to that found in *Solenopsis invicta* Buren (Hymenoptera: Formicidae) and *Apis mellifera* L. (Hymenoptera: Apidae) (Supplementary Table S2) as reported by Rahman et al. (2009) and Veenstra (2010). Moreover, Lom-CAPA contained CAPA-1, CAPA-2, and DH-1 segments along with a PK segment in the C-terminal. These results are similar to *Aedes aegypti* L. (Diptera: Culicidae), *Tribolium castaneum* Herbst (Coleoptera: Tenebrionidae), *Athalia rosae* L. (Hymenoptera: Tenthredinidae) (Supplementary Table S2). In *L. migratoria*, we found three types of pyrokinins, including Lom-CAPA, Lom-PBAN, and Lom-DH. *Teleogryllus commodus* Walker (Orthoptera: Gryllidae), *Zootermopsis nevadensis* Hagen (Isoptera: Termopsidae), and *Blattella germanica* L. (Blattodea: Blattellidae) also has novel *dh* genes with eight, six, and 23 DH peptide copies respectively (Supplementary Table S2). On the basis of these data, the CAPA and PBAN of insects have a fixed structure, but one of them may lose the DH segment, and the novel *dh* genes that we have found in this study contained multiple DH copies (Supplementary Table S2). As the DH functions were not properly documented in previous studies, we considered that at least one of the *capa*, *pban*, or *dh* genes was involved with the DH function. This was vital for insect physiological metabolism. The duplicate *dh* genes and multiple copies of DH in the DH precursors in Orthoptera may be involved with diapause regulation (Supplementary Table S2). The differentiation of *Lom-dh* with tandem DHs permits the locust to exploit its environment to maximize offspring survival. *L. migratoria*, along with termites and cockroaches are important pests. Understanding the function of novel *dh* genes in the physiological metabolism of these taxa might be helpful for developing new pest control methods.

### Novel *Lom-dh* Genes Can Encode Multiple DH Neuropeptides to Induce Locust Egg Diapause

We found that type I DH-like neuropeptides from novel *Lom-dh* genes can induce locust diapause (Table 1). Previous studies on diapause in the silkworm *B. mori* showed that, depending on the neuropeptide, doses ranging from 1 pmol to 20 nmol per individual induced embryonic diapause (Sato et al., 1993). Moreover, the locust pyrokinin Lom-PK is threefold more active than native Bom-DH as a diapause induction agent at the dose of 20–4,000 pmol/individual (Nachman et al., 1993). Injection of 200 pmol SDH1 induced locust diapause at a rate higher than that found when locusts were injected with 2,000 pmol LDH1. This result may relate to the N-terminal hydrophobic structure, as LDH1 contains LWFGPRI (Zhang et al., 2015). For both LDH2 and SDH2, diapause induction occurred only at doses of at least 10^4^ pmol. To increase the induction of diapause using LDH1 and SDH1 beyond 50%, the concentration of doses needed to be increased. Neuropeptides working in combination with receptors are concentration dependent, with the relationship showing evidence of coevolution (Park et al., 2002; Grimmelikhuijzen and Hauser, 2012). Thus, the DH receptor recognition of DH 1 and DH 2 may be caused by the difference in the last amino acid between LWFGPRV and LWFGPRI. This study may aid in the development of other DH analogs and antagonists that could disrupt insect diapause and be used as pest management tools. Insect CAPA neuropeptides may be associated with cold tolerance, but no previous data have indicated that it was involved in locust diapause (Xu and Denlinger, 2003; Terhzaz et al., 2015). DH associated with the *pban* gene is known to be involved in insect diapause regulation (Jurenka and Nusawardani, 2011). No DH produced by the *pba*n gene in some diapausing insects, such as *L. migratoria*; this suggests that the DH, in these species, may be derived from *capa* or *dh* genes.

To verify the effect of *Lom-dh* genes on locust diapause at the mRNA level, we injected ds*Lom-dh1*, ds*Lom-dh2/3*, ds*Lom-dh4/5*, and ds*Lom-dhall* to implement RNA interference on all *Lom-dh* genes. Because of the high similarity between *Lom-dh2* and *Lom-dh3*, *Lom-dh4* and *Lom-dh5*, the same dsRNA (ds*Lom-dh2/3*, ds*Lom-dh4/5*) were used to implement RNA interference on all of them. Moreover, ds*Lom-dhall*, which is composed of equimolar amounts of ds*Lom-dh1*, ds*Lom-dh2/3*, and ds*Lom-dh4/5*, was used to implement RNAi on all *Lom-dh* genes simultaneously. The interference efficiency test showed that relative mRNA levels were significantly decreased after injection of ds*Lom-dhall* under both long and short photoperiods. This result proved that five different *Lom-dh* genes are susceptible to RNA interference by simultaneous injection of three dsRNAs in *L. migratoria*. The results demonstrate that the diapause incidences of locust eggs are significantly (*p* < 0.05) decreased by RNA interference of *Lom-dh1* to *Lom-dh5* genes of adult females under short photoperiod. Among these, the ds*Lom-dh4/5* treatment was the most effective. We did not observe any effects by injecting any ds*Lom-dh* under a long photoperiod. Results showed that the expression level of *Lom-dh* genes were higher under short photoperiod compared to long photoperiod (Figure 4). These findings are consistent with the results obtained from RNAi.

### DH Peptides and *Lom-dh* Only Have Function Under Short Photoperiod

As early as 1957, the existence of a DH from *B. mori* maternal subesophageal ganglions (SG) was suggested to act on the developing ovaries via the hemolymph, thereby causing the entry of eggs into diapause (Hasegawa, 1957). Interestingly, previous research found that DH was not capable of inducing pupal diapause in the *Helicoverpa/Heliothis* complex, but instead it effectively terminates pupal diapause, a surprising contrast to the results noted in *B. mori* (Xu and Denlinger, 2003; Zhang et al., 2004; Zhao et al., 2004). In previous studies, the functions of DH on diapause regulation haven’t been found in any other insect orders except Lepidoptera. Our work is the first evidence that diapause of *L. migratoria*, an Orthoptera insect, can be induced by DH. Our results are consistent with the observations on *B. mori*, but are in contrast to the results with the *Helicoverpa/Heliothis* complex. Diapause induction of both *L. migratoria* and *B. mori* is a trans-generation process. In the *Helicoverpa/Heliothis* complex, diapause occurs at a fixed developmental stage (pupal stage). This may be the probable reason to explain why DH functions are so different in diapause regulating. However, we cannot confirm why the sequences of these DHs are almost similar, yet their functions in diapause regulation are opposite. The specific mechanism of variable function of DH still remains an interesting and hot topic for future study.

To investigate how photoperiod and DH affect *L. migratoria* diapause, two important pathways of diapause regulation, i.e., insulin signaling pathway and MAPK signaling pathway, have been described below. Circadian clock oscillations function in the insect brain or peripheral nerve and likely contribute to photoperiodism (Ito et al., 2008; Ikeno et al., 2010). The circadian genes are linked to insulin signaling, which is the most important regulator of normal growth in insects and leads to the production of juvenile hormone through the PI3-kinase/Akt pathway. Then, forkhead box protein O (FOXO) is phosphorylated and it blocks the translocation protein into the nucleus under long-day photoperiod (Lee et al., 2001; Jünger et al., 2003; Sim and Denlinger, 2008, 2013). Suppression of insulin signaling causes the FOXO, translocated into the nucleus, to initiate transcription of downstream genes and induce diapause in *D. melanogaste*r, *C. pipiens*, and *C. elegan*s under short photoperiod (Gerisch et al., 2001; Williams et al., 2006; Sim and Denlinger, 2008). Previous study in silkworm *B. mori* revealed that DH activated the DH receptor signals to MAPK signaling pathway through Gq-PLC-PKC-dependent cascade (Jiang et al., 2016). In this study, the DH peptides and dsRNA (ds*Lom-dh*) that were injected, at various concentrations, did not affect *L. migratoria* diapause incidence under long photoperiod. This suggests that there may be a cross talk between insulin signaling pathway and DH activated MAPK signaling pathway in diapause induction of *L. migratoria* under long photoperiod where the insulin signaling dominated the diapause regulation and subsequently, the DH activated MAPK signaling was blocked. On the contrary, insulin signaling pathway was inhibited under short photoperiod, thus, the DH activated MAPK signaling pathway takes over, leading to diapause induction. Hence, we suggested that the DH function may be inhibited by insulin signaling pathway under long photoperiod.

Lepidoptera studies have demonstrated that PBAN, subesophageal ganglion neuropeptide (SGNP), and other pyrokinin family neuropeptides have functions similar to DH, but they are less effective than DH in diapause termination in *Helicoverpa armigera* and *Helicoverpa assulta* (Zhang et al., 2004; Zhao et al., 2004). All of these pyrokinin family neuropeptides have consensus sequences and could competitively bind to the same GPCR. In addition to encoding I–V type DH-like neuropeptides, the *Lom-dh* genes can also encode other neuropeptides, such as KGLVASARVa, which may competitively bind DH receptors. Many neuropeptides may exist in *L. migratoria* to compete with DH-like neuropeptides for DH receptors under long-day photoperiod. Therefore, maybe not all of the DH-like neuropeptides play as an active role in regulating diapause. Our study verified the function of only type I DH. The functions of II–V type DH and other neuropeptides encoded by *Lom-dh* genes in diapause regulation will require further study.

## Conclusion

The diapause hormone, only encoded by *capa* or *pban*, and its role in insect diapause regulation, is well researched. In this study, a new *capa*/*pban* family member, *dh* is reported for the first time in *L. migratoria*. We also identified novel DH neuropeptides. Characters of *dh* genes are as follows: (1) there is no intron in *dh* genes. (2) It contains tandem DH-like neuropeptide repeats. (3) Compared to *capa* and *pban*, these *dh* genes have codon bias and they belong to a new type of genes in phylogeny. Finally, the diapause induction function of *dh* genes was identified by RNAi technology. Moreover, functions of DH neuropeptides encoded by these *dh* genes were also identified by injection bioassay. Our study shows that multiple DH neuropeptides regulate locust diapause. Diapause regulation by DH in locusts maybe more likely a quantitative character rather than a qualitative character. Our study provides a new perspective for insect diapause regulation. However, how *Locusta* and other species evolved this new type of genes and their importance to insect physiology still needs to be explored.

## Materials and Methods

### Insects

The *L. migratoria* colony studied was maintained at the Institute of Plant Protection, CAAS laboratory for 8 years. Third instars nymphs were collected from the rearing cages and transferred to 20 cm × 20 cm × 28 cm mesh cages in artificial climate chambers (PRX-250B-30, Haishu Saifu Experimental Instrument Factory, Ningbo, China) and reared to adults at 16:8 L:D photoperiod, 60% RH, 28°C conditions to produce non-diapause eggs and at 10:14 L:D photoperiod, 60% RH, 28°C conditions to induce diapause eggs.

Eggs were laid in sand beneath the cages and were collected at an interval of 2 days. Eggs were then transferred into plastic Petri dishes (90 mm × 50 mm) and incubated on vermiculite at 28°C, 60% RH and a 10:14 L:D photoperiod. Nymphs were fed glasshouse grown wheat plants at 16:8 L:D photoperiod, 60% RH and 28°C with food replaced two to three times on daily basis until they reached the adult stage.

To investigate the basis for DH precursor sequences and function, we dissected five adult females from each photoperiod treatment to remove the brain ganglia and nerve cord. The tissues from each locust in each treatment were transferred to 1.5 ml Eppendorf tubes before being snap-frozen in liquid nitrogen and stored at ‑80°C for subsequent gene amplification (see below).

### *pban/capa* Family Gene Sequences Analysis

The insect CAPA/PBAN protein precursor sequences were downloaded from the National Center for Biotechnology Information (NCBI) protein database^1^. Lom-PK peptides were downloaded from NCBI and merged together as a hypothetical precursor. The *L. migratoria* whole genome sequences contigs were downloaded in fasta from NCBI Whole Genome Shotgun website^2^ and analyzed by local BLAST on a desktop PC.

*Locusta migratoria* genome sequences were used to build searchable BLAST databases^3^ using ncbi-blast-2.2.30+. Later on, the CAPA/PBAN proteins were aligned to translate locust genome (Camacho et al., 2009). Alignments longer than 100 bp were retained as candidate sequences for identifying the target genes (Supplementary Figure S5).

### DNA and cDNA Cloning of the Target Genes

Frozen tissue collected from adult locusts was macerated in 1 × TE buffer before extraction. Genome DNA was extracted using a TaKaRa MiniBEST Universal Genomic DNA Extraction Kit and diluted by ddH_2_O at a 1:10 ratio. Total RNA was extracted using Invitrogen TRIzol and purified between 1.9 and 2.2 (A260/A280). cDNA was reverse-transcribed using M-MLV reverse transcriptase and recombinant RNase inhibitor (TaKaRa) and diluted by ddH_2_O at a 1:4 ratio. All DNA and cDNA were prepared for gene amplification. Gene-specific primers were designed according to the predicted *pban*/*capa* gene sequences and synthesized by SANGON (Beijing, China). Specific primers were designed to obtain the fragment of the target genes using polymerase chain reaction (PCR; Supplementary Table S3). PCR procedures were performed as follows: 95°C 3 min; 36 cycles of 95°C 30 s; 58°C 30 s; 72°C 90 s; and 72°C 10 min. Bands of interest were gel purified and cloned using the pGEM T-Easy Vector System (Promega) per the manufacturer’s instructions, and then sequenced by Invitrogen, Shanghai, China (Li et al., 2013).

### Phylogenetic Analysis of CAPA/PBAN Precursors in Insecta

The evolutionary history was inferred using the Neighbor-Joining method (Saitou and Nei, 1987). The optimal tree with the sum of branch lengths = 29.94781313 is shown (Supplementary Figure S2). The percentage of replicate trees in which the associated taxa clustered together in the bootstrap test (1,000 replicates) is shown next to the branches (Felsenstein, 1985) (Supplementary Figure S2). The tree is drawn to scale, with branch lengths in the same units as those of the evolutionary distances used to infer the phylogenetic tree. The evolutionary distances were computed using the Poisson correction method (Zuckerkandl and Pauling, 1965) and are in units of the number of amino acid substitutions per site. The analysis involved 94 amino acid sequences. We removed all ambiguous positions for each sequence pair. The final data-set had a total of 737 positions. Evolutionary analyses were conducted in MEGA6 (Tamura et al., 2013).

### Codon Bias Analysis

Codon bias analysis was calculated using CodonW, version 1.4.2 (Peden, 2000). Codon Adaptation Index (CAI) (Sharp and Li, 1987), Frequency of Optimal codons (FOP) (Ikemura, 1981), and Codon Bias Index (CBI) (Bennetzen and Hall, 1982). The G+C content of the third position of synonymous codons (GC3s) was used to evaluate codon bias.

### *Lom-capa/pban/dh* Genes Structure Analysis

For subsequent sequence analysis, the open reading frame (ORF) for *Lom-capa/pban* genes in *L. migratoria* genomic DNA was predicted using the website^4^. Signal peptides were predicted using the hidden Markov model of SignalP4.1^5^ (Veenstra, 2000). Putative cleavage sites in the precursors were analyzed as described by Veenstra (2000) or using web-based NeuroPred program^6^.

We conducted evolutionary analyses of pyrokinin consensus sequences in MEGA6 (Tamura et al., 2013). The evolutionary history was inferred using the maximum likelihood method based on the Poisson correction model (Zuckerkandl and Pauling, 1965). The tree with the highest log likelihood (−57000.9915) is shown (Figure 2). The percentage of trees in which the associated taxa clustered together is shown next to the branches (Figure 2). Initial tree(s) for the heuristic search were obtained by applying the Neighbor-Joining method to a matrix of pairwise distances estimated using a JTT model. The tree is drawn to scale, with branch lengths measured in the number of substitutions per site. The analysis involved 16 amino acid sequences. There were a total of seven positions in the final data set.

### Liquid Chromatography–Tandem Mass Spectrometry Analysis and Neuropeptide Identification

The central neuron systems of *L. migratoria* under both long (L:D 16:8) and short (L:D 10:14) photoperiods at a constant temperature of 28°C were homogenized and neuropeptides were extracted at 4°C using a 90:9:1 solution of methanol, H_2_O, and acetic acid to extract the neuropeptides. The homogenate was then centrifuged at 12,000 × *g* for 10 min at 4°C. The supernatant was collected and vacuum-dried using a SpeedVac system (RVC 2–18, Marin Christ, Osterod, Germany) to obtain a neuropeptide sample. Samples were stored at ‑80°C before liquid chromatography–tandem mass spectrometry (LC-MS/MS) analysis.

Neuropeptide pellets were re-dissolved in 0.1% formic acid in distilled water, and the final peptide concentration was quantified using a Bradford assay. LC-MS/MS analysis was performed on the Easy-nLC 1000 (Thermo Fisher Scientific, Bremen, Germany) coupled with the LTQ-Orbitrap Elite (Thermo Fisher Scientific) hybrid mass spectrometer with three replicates. Samples were loaded onto a 2 cm long, 100 μm inner diameter fused silica trap column containing 5.0 μm Aqua C18 beads (Thermo Fisher Scientific) for 2 min in buffer A (0.1% formic acid) at a flow rate of 5 μL/min before analytical separation. Peptides were separated on a column packed with 2 μm C18 (100 Å, 75 μm × 50 cm, Thermo Fisher Scientific) at a flow rate of 350 nL/min using the following gradients: from 3 to 8% buffer B in 5 min, from 8 to 20% buffer B in 80 min, from 20 to 30% buffer B in 20 min, from 30 to 90% buffer B in 5 min, and 90% buffer B in 10 min. In data-dependent acquisition mode (range from 300 to 1800 m/z with a resolution of 70,000 at 400 m/z), the 10 most abundant precursor ions with charge states greater than +1 were fragmented and previously acquired precursor ions (repeat count 1, repeat duration: 30 s; exclusion duration 45 s) were dynamically excluded. MS/MS spectra were acquired in higher energy collisional dissociation (HCD) mode with a resolution of 17,500 at 400 m/z and started from 100 m/z using a normalized energy of 30. The MS/MS data were acquired in raw files using Xcalibur software (version 2.2, Thermo Fisher Scientific).

The extracted MS/MS spectra were searched against a composite database containing 3,090 protein sequences of *L. migratoria* using in-house PEAKS software (version 7.0, Bioinformatics Solutions, Waterloo, ON, Canada). The following modifications were applied: C-terminal amidation (*A*, −0.98) and pyroglutamination from Q (*P*, −17.03). The other parameters used were the following: parent ion mass tolerance, 15.0 ppm; fragment ion mass tolerance, 0.05 Da; enzyme, none; and maximum allowed variable post-translational modification per peptide, 2. A fusion target-decoy approach was used for the estimation of the false discovery rate and controlled at ≤1.0% (−10 log *P* ≥ 20.0) at both protein and peptide levels. Neuropeptide identifications were used only if at least two spectra were identified in one sample.

### Bioassay of Neuropeptides Activity

Based on the Lom-DH2 amino acid sequence, four neuropeptides were synthesized (SANGON, Shanghai, China). The neuropeptides were designated as follows: LDH1: MHSKNVVVQRCIQTGFSDGGSIRNVPETSLWFGPRIa, purity > 93.06%; SDH1: LWFGPRIa, purity > 96.27%; LDH2: SNPETNLWFGPRVa, purity > 99.11%; SDH2: LWFGPRVa, purity > 99.10%. For neuropeptide activity bioassays, solutions (10 μl/individual) containing different doses (20–20,000 pmol/individual) of the four neuropeptides (LDH1, SDH1, LDH2, and SDH2) were micro-injected [microprocessor Controller PAX100-3, microapplicator (Burkard, United Kingdom)] into the interstitial membrane between the second and third abdominal segments of the insects with a microapplicator. We randomly removed a total of 100 fifth instar females from the four cages in each photoperiod treatment and injected them with one of the four neuropeptides to provide 25 individuals per treatment. The selected fifth instar nymphs were almost at the verge of adulthood. Another 25 females taken from each photoperiod treatment were injected with distilled water as the controls.

Following treatment, locusts were moved to new mesh cages (20 cm × 20 cm × 28 cm) and provided with bouquets of glasshouse grown wheat. At the same time, 25 adult males were introduced to the cages before being returned to the allocated photoperiod treatments from which they had been collected. The floor of the cages was covered by a 2 cm layer of sieved sterilized sand, with the cage conditions maintained until egg laying commenced. Generally, the time lapse between injection of DH and egg laying was 2–3 weeks. Once oviposition was observed, eggs were collected, using a camel paint brush, after every 48 h for 10 days and transferred to Petri dishes lined with moist filter paper, before transfer to 28°C and 60% RH to slow development. Eggs were kept at 28°C for 30 days until eclosion of first instar nymphs ended. To account for non-viable eggs, remnant unhatched eggs were kept at 4°C for 60 days to provide time to break diapause after which they were incubated at 28°C for 30 days and any further first instar emergence was recorded. The diapause incidence was calculated as following: Diapause incidence (%) = H2/(H1+H2), where H1 are individuals emerging within 30 days at 28°C and H2 individuals emerging following chilling at 4°C for 60 days.

### RNA Interference Treatment by Double-Stranded RNA Injection and Real-Time PCR

The dsRNA was generated by *in vitro* transcription using the T7 RiboMAX system (Promega) according to the manufacturer’s protocol. Templates for *in vitro* transcription reactions were prepared by PCR amplification from plasmid DNA of the cDNA clone of target genes using the primer pairs with a T7 polymerase promoter sequence at 5′-end (Supplementary Table S3). A total of 10 μl of dsRNAs (1 μg/μl) for the target genes, GFP controls, or water controls were injected into the ventral area between second and third abdominal segments of female adults within 3 days after the nymph to adult molt. The ds*Lom-dhall* was composed of ds*Lom-dh1*, ds*Lom-dh2/3*, and ds*Lom-dh4/5* with a mole ratio of 1:1:1. For each gene, 75 nymphs were injected and divided into three groups. The effects of RNAi on the mRNA levels were investigated by quantitative real-time PCR (qRT-PCR) at 48 h after injection. To monitor the transcript levels of the target genes, total RNA was extracted from whole bodies. For each target gene, three individuals from each group were used for RNA extraction. The diapause incidence was observed according to previous section of bioassay of neuropeptides activity. Each treatment included 25 female adults that were injected and their eggs were divided into three groups. The effect of RNAi on mRNA levels and diapause incidence was monitored as described earlier.

We used the same methods of RNA extraction and reverse-transcription as described earlier. To quantify the gene expression, the 2^–ΔΔCt^ method was used, with β-*actin* as the positive control and for normalizing the data. The specific primers used were for qRT-PCR (Supplementary Table S3). We performed RT-PCR with the SYBR Premix ExTaq™ (TaKaRa, Dalian, China) on the ABI 7500 Real-Time PCR System (Applied Biosystems, Foster City, CA, United States). The β-*actin* gene was used as an internal control. The reaction was performed using the following conditions: denaturation at 95 ration 60 s, followed by 40 cycles of amplification (95°C, 15 s; 60°C, 60 s). Melting curve analysis was performed to confirm the specificity of amplification. Each plate was repeated three times in independent runs for all reference and selected genes.

### Statistical Analysis

We compared the differences between treatments either by Student’s *t*-test or by one-way analysis of variance (ANOVA) followed by a Tukey’s test for multiple comparisons. We considered differences significant at *P* < 0.05 and reported values as mean ± SE. We analyzed data using the SPSS software (version 15.0; SPSS Inc., Chicago, IL, United States).

## Author Contributions

ZZ and KH conceived and designed the study. KH performed the experiments, data collection and analysis, and drafted the manuscript. HU, XT, and MM reviewed and edited the manuscript. All authors approved the final manuscript.

## Conflict of Interest Statement

The authors declare that the research was conducted in the absence of any commercial or financial relationships that could be construed as a potential conflict of interest.

## Footnotes

1 http://www.ncbi.nlm.nih.gov/protein/

2 http://www.ncbi.nlm.nih.gov/Traces/wgs/?val=AVCP01#contigs

3 ftp://ftp.ncbi.nlm.nih.gov/blast/executables/blast+/LATEST/

4 https://omictools.com/genscan-tool

5 http://www.cbs.dtu.dk/services/SignalP/

6 http://stagbeetle.animal.uiuc.edu/cgi-bin/neuropred.py

## Supplementary Material

FIGURE S1 The seven predicted PBAN, CAPA, tryptopyrokinin precursors, and one pyrokinin amino acid sequences in *Locusta migratoria* L. Blue sequences indicate signal peptide, green sequences indicate endoproteolytic cleavage sites, and yellow sequences indicates short neuropeptides.

FIGURE S2 Phylogenetic analysis of CAPA/PBAN precursors in Insecta. Circles represent Hymenoptera, squares represent Lepidoptera, upward triangles represent Diptera, diamonds represent CAPA and PBAN of *Locust migratoria*, and downward triangles represent TPK of *Locusta migratoria.*

FIGURE S3 Codon bias analysis of *L. migratoria capa*, *pban* and *TPK* genes. The lowercase a, b, and c indicated that statistically significant difference of different genes was considered on an error probability of *p* < 0.05 by one-way ANOVA using Duncan’s multiple-range test.

FIGURE S4 The sequence logo was created using Weblogo (Crooks et al., 2004).

FIGURE S5 R mapping result of PBAN/PK precursors based on blasting *Locusta migratoria* L. genome. The horizontal coordinate indicates genome files, whereas the vertical coordinate indicates mapped amino acid numbers; light blue to dark blue indicates e-value expression level.

TABLE S1 Candidate *pban/capa* family gene sequences of the *L. migratoria* genome contigs.

TABLE S2 PBAN, CAPA, and DH precursors of insects. The black box indicates consensus sequences, the green amino acid sequence indicates endoproteolytic cleavage sites, and the blue amino acid indicates DH neuropeptides.

TABLE S3 Specific primers of this chapter.
