# Supplementary figures and images for: Novel Lom-dh Genes Play Potential Role in Promoting Egg Diapause of Locusta migratoria L
Source: Front Physiol. 2019 Jun 18;10:767. doi: 10.3389/fphys.2019.00767 (PMC6591537; doi:10.3389/fphys.2019.00767)

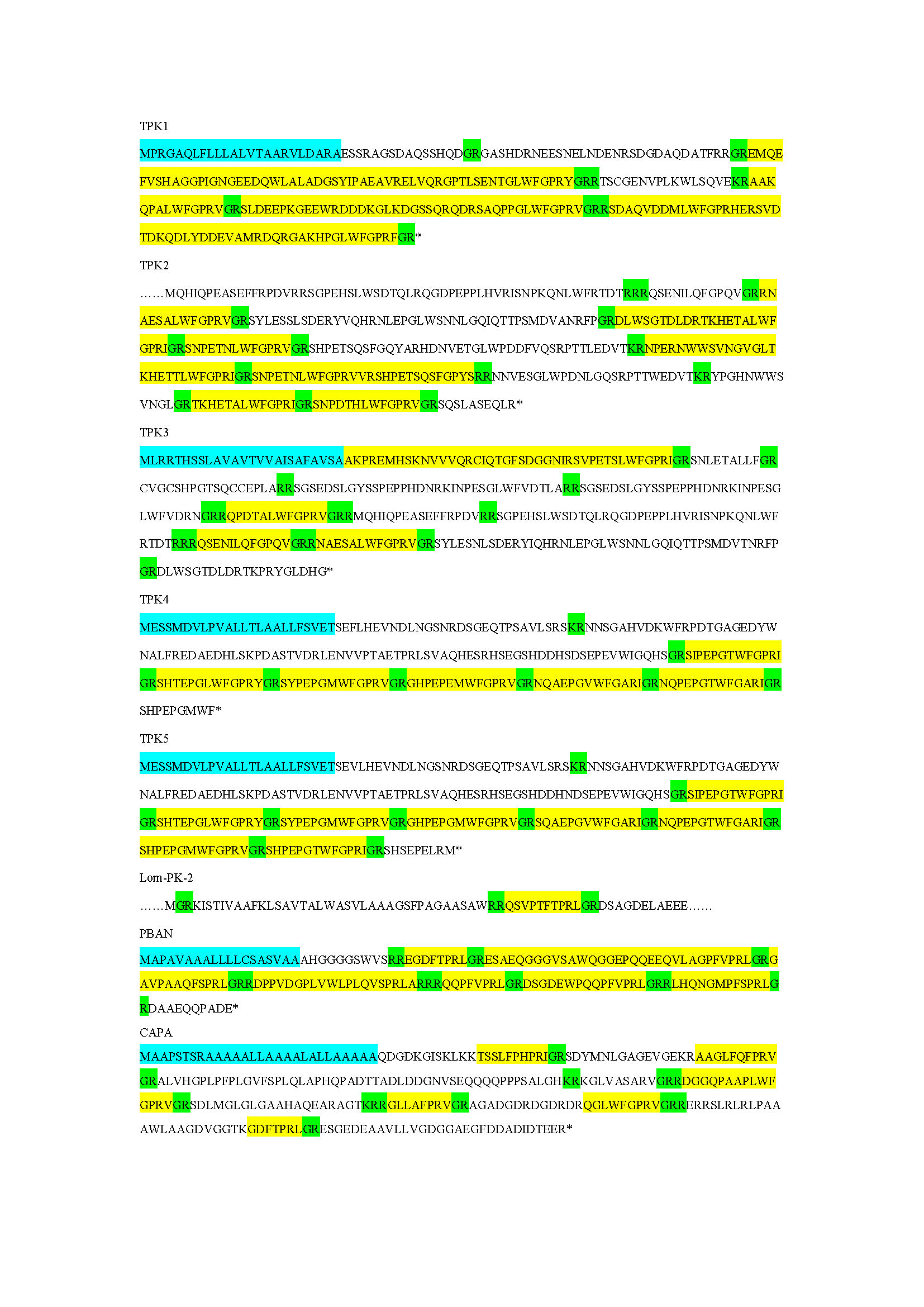

Supplement: FIGURE S1 — The seven predicted PBAN, CAPA, tryptopyrokinin precursors, and one pyrokinin amino acid sequences in Locusta migratoria L. Blue sequences indicate signal peptide, green sequences indicate endoproteolytic cleavage sites, and yellow sequences indicates short neuropeptides. [file Image_1.JPEG]

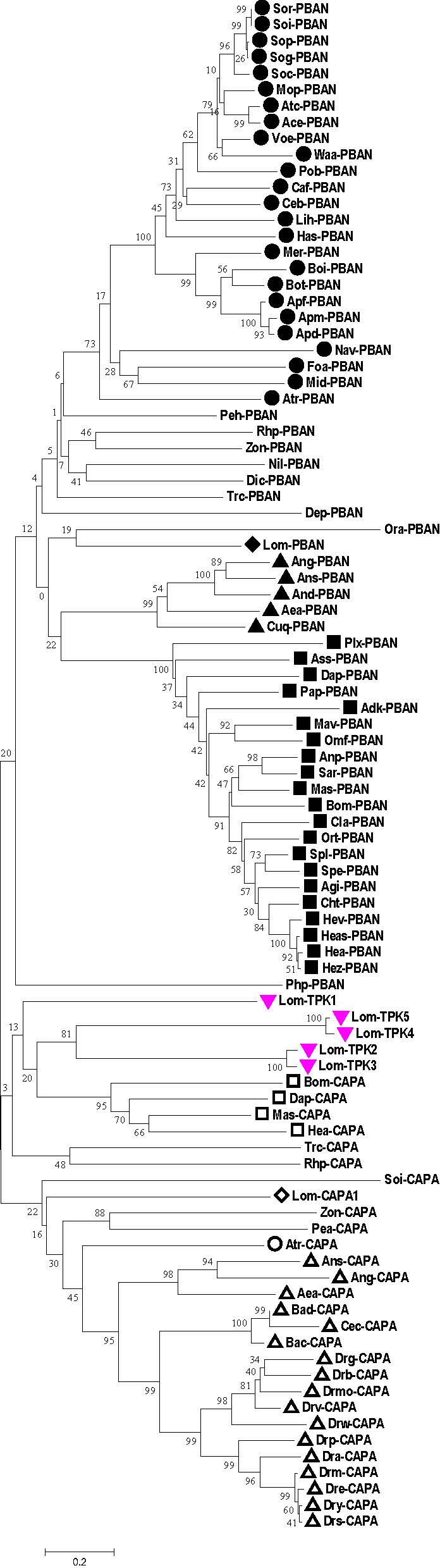

Supplement: FIGURE S2 — Phylogenetic analysis of CAPA/PBAN precursors in Insecta. Circles represent Hymenoptera, squares represent Lepidoptera, upward triangles represent Diptera, diamonds represent CAPA and PBAN of Locust migratoria, and downward triangles represent TPK of Locusta migratoria. [file Image_2.JPEG]

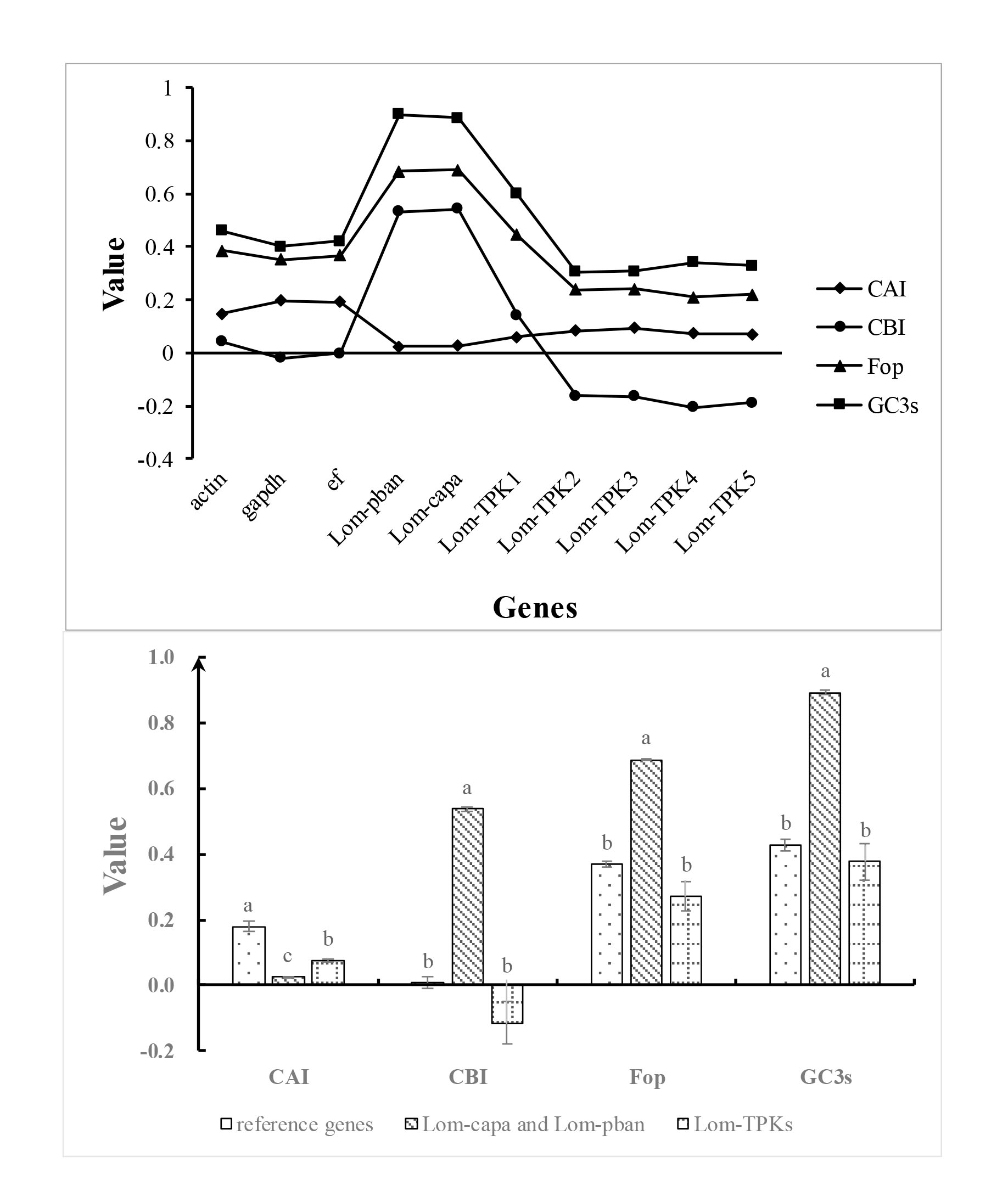

Supplement: FIGURE S3 — Codon bias analysis of L. migratoria capa, pban and TPK genes. The lowercase a, b, and c indicated that statistically significant difference of different genes was considered on an error probability of p < 0.05 by one-way ANOVA using Duncan’s multiple-range test. [file Image_3.JPEG]

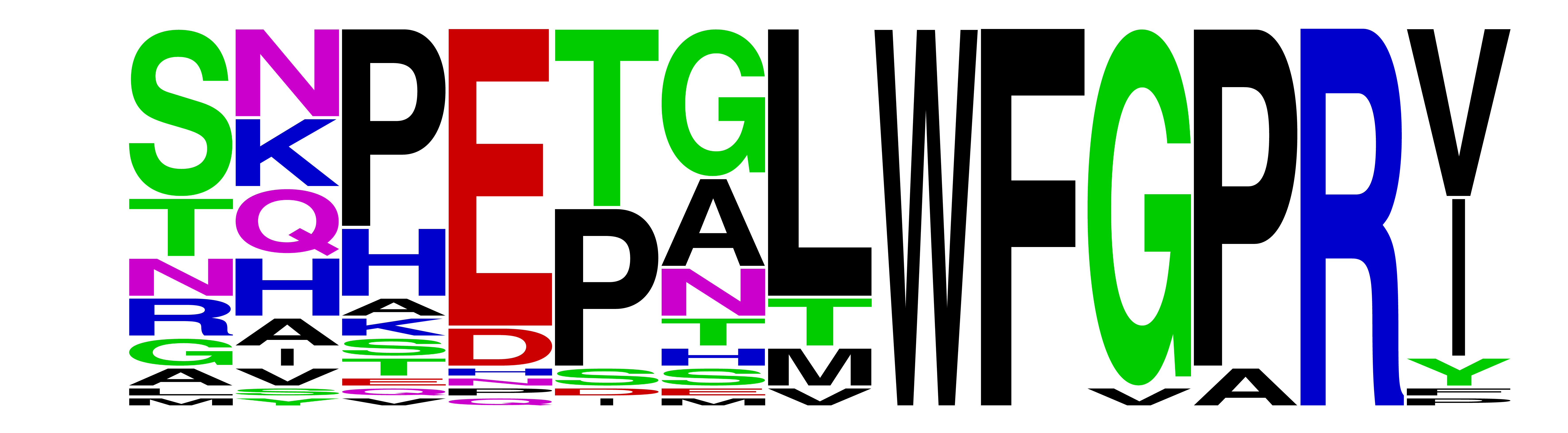

Supplement: FIGURE S4 — The sequence logo was created using Weblogo (Crooks et al., 2004). [file Image_4.PNG]

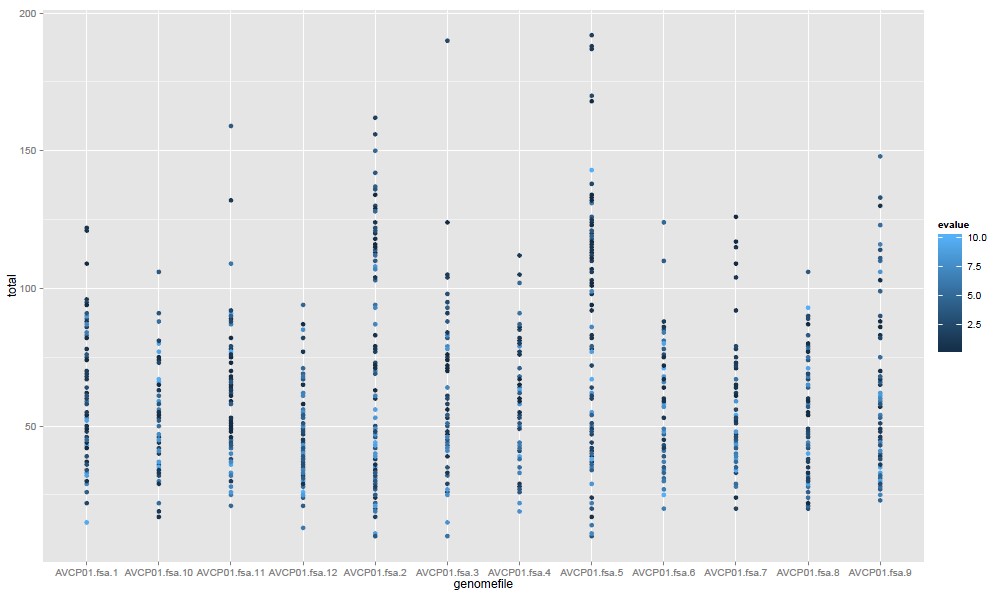

Supplement: FIGURE S5 — R mapping result of PBAN/PK precursors based on blasting Locusta migratoria L. genome. The horizontal coordinate indicates genome files, whereas the vertical coordinate indicates mapped amino acid numbers; light blue to dark blue indicates e-value expression level. [file Image_5.JPEG]
