# Supplementary material for: Novel Lom-dh Genes Play Potential Role in Promoting Egg Diapause of Locusta migratoria L
Source: Front Physiol. 2019 Jun 18;10:767. doi: 10.3389/fphys.2019.00767 (PMC6591537; doi:10.3389/fphys.2019.00767)
Supplement: TABLE S1 — Candidate pban/capa family gene sequences of the L. migratoria genome contigs. [file Table_1.DOCX]

**Table S1 Candidate *pban/capa* gene sequences of *Locusta migratoria* L. genome contigs**

| Query | Database | Total | Genome file |
| --- | --- | --- | --- |
| gi\|751776732\|ref\|XP_011197340.1\| | gb\|AVCP010037508.1\| | 122 | AVCP01.fsa.1 |
| gi\|310616640\|tpg\|DAA33896.1\| | gb\|AVCP010060505.1\| | 109 | AVCP01.fsa.1 |
| gi\|752879401\|ref\|XP_011257226.1\| | gb\|AVCP010159522.1\|(TPK4) | 103 | AVCP01.fsa.2 |
| gi\|500862\|dbj\|BAA03755.1\| | gb\|AVCP010159529.1\|(TPK3) | 162 | AVCP01.fsa.2 |
| gi\|478261721\|gb\|AGI96545.1\| | gb\|AVCP010201651.1\| | 105 | AVCP01.fsa.3 |
| gi\|347810662\|gb\|AEP25400.1\| | gb\|AVCP010272810.1\| | 190 | AVCP01.fsa.3 |
| gi\|728891537\|gb\|AIY99907.1\| | gb\|AVCP010299128.1\| | 112 | AVCP01.fsa.4 |
| gi\|3341394\|emb\|CAA08774.1\| | gb\|AVCP010303494.1\| | 102 | AVCP01.fsa.4 |
| gi\|746851984\|ref\|XP_011056531.1\| | gb\|AVCP010343123.1\| | 112 | AVCP01.fsa.4 |
| gi\|30984070\|gb\|AAP41132.1\| | gb\|AVCP010410303.1\|(TPK2) | 192 | AVCP01.fsa.5 |
| gi\|27657760\|gb\|AAO18192.1\| | gb\|AVCP010410310.1\|(TPK5) | 168 | AVCP01.fsa.5 |
| gi\|768425305\|ref\|XP_011553647.1\| | gb\|AVCP010480295.1\| | 102 | AVCP01.fsa.5 |
| gi\|815806702\|ref\|XP_012224133.1\| | gb\|AVCP010538049.1\| | 110 | AVCP01.fsa.6 |
| gi\|815806702\|ref\|XP_012224133.1\| | gb\|AVCP010586161.1\| | 124 | AVCP01.fsa.6 |
| gi\|768425305\|ref\|XP_011553647.1\| | gb\|AVCP010629157.1\| | 126 | AVCP01.fsa.7 |
| gi\|646707069\|gb\|KDR13989.1\| | gb\|AVCP010654946.1\| | 115 | AVCP01.fsa.7 |
| gi\|815806702\|ref\|XP_012224133.1\| | gb\|AVCP010797131.1\| | 106 | AVCP01.fsa.8 |
| gi\|357611058\|gb\|EHJ67284.1\| | gb\|AVCP010817043.1\| | 111 | AVCP01.fsa.9 |
| gi\|194142651\|gb\|EDW59054.1\| | gb\|AVCP010869438.1\| | 103 | AVCP01.fsa.9 |
| gi\|194142651\|gb\|EDW59054.1\| | gb\|AVCP010869439.1\| | 103 | AVCP01.fsa.9 |
| gi\|74800397\|sp\|Q7PTL2.2\|PBAN_ANOGA | gb\|AVCP010875633.1\| | 130 | AVCP01.fsa.9 |
| gi\|62766017\|gb\|AAX99220.1\| | gb\|AVCP010882506.1\|(TPK1) | 148 | AVCP01.fsa.9 |
| gi\|357611058\|gb\|EHJ67284.1\| | gb\|AVCP010991511.1\| | 106 | AVCP01.fsa.10 |
| gi\|751776732\|ref\|XP_011197340.1\| | gb\|AVCP011074633.1\| | 109 | AVCP01.fsa.11 |
| gi\|466524\|dbj\|BAA05954.1\| | gb\|AVCP011075735.1\| | 159 | AVCP01.fsa.11 |
| gi\|728891537\|gb\|AIY99907.1\| | gb\|AVCP011104938.1\| | 132 | AVCP01.fsa.11 |
| gi\|307179436\|gb\|EFN67760.1\| | gb\|AVCP010159522.1\| | 103 | AVCP01.fsa.2 |
| Lom-PK-2 | gb\|AVCP010267064.1\| | 10 | AVCP01.fsa.3 |
| gb\|GCGJ01044456.1\|(Lom-pban) | gb\|AVCP011098992.1\| | | AVCP01.fsa.11 |
|  | gb\|AVCP011098990.1\| | |  |
| gb\|GCGJ01004763.1\|(Lom-capa) | gb\|AVCP011143372.1\| | | AVCP01.fsa.12 |
|  | gb\|AVCP011143377.1\| | |  |
|  | gb\|AVCP011143364.1\| | |  |
|  | gb\|AVCP011143367.1\| | |  |
