# Supplementary material for: Novel Lom-dh Genes Play Potential Role in Promoting Egg Diapause of Locusta migratoria L
Source: Front Physiol. 2019 Jun 18;10:767. doi: 10.3389/fphys.2019.00767 (PMC6591537; doi:10.3389/fphys.2019.00767)
Supplement: TABLE S2 — PBAN, CAPA, and DH precursors of insects. The black box indicates consensus sequences, the green amino acid sequence indicates endoproteolytic cleavage sites, and the blue amino acid indicates DH neuropeptides. [file Table_2.DOCX]

| **Table S2 PBAN, CAPA and DH precursors of insects. Black-box indicates consensus sequences, green amino acid sequence indicates endoproteolytic cleavage sites, blue amino acid indicates diapause hormone neuropeptides.** |
| --- |
| >gi\|752507684\|ref\|NP_001291527.1\| PBAN-type neuropeptides-like precursor [*Solenopsis invicta*]  MIVTRNSVNRATIVCIMAMLLCLGSRASGEYESREIGSNGGSSESRSPSNDFGSCIDGKCIKRTSQDIASGMWFGPRLGKRYKSDEKQELSSEIEILANALDGVRWAVITIPASDKRQPQFTPRLGRGSGEDLSYGDAYEVDEDDHPLFVPRLGRRLPWIPSPRLGRQLRNVLRKL |
| >gi\|662036813\|gb\|AIE44140.1\| capa-long isoform [*Solenopsis invicta*]  MQDNRFFIFVILLAFSTSLNLARCSVGQNYEPTREGQKLKINDRRSAGLVAYPRIGRKSDLFPRLGRTFGIIQKPRVGRSDDSSLGDLNRLHDLPADTDIEFYITRDMEPDVLLNFDYEDYADKPIAFKHADKIQKDDSWLMPDHVRGYKDPRFAQKIDDLRSYYSILRGSRNSQGQGGYTPRLGRESEHDAANFP |
| >gi\|160420323\|ref\|NP_001104182.1\| PBAN-type neuropeptides precursor [*Apis mellifera*]  MIGFAVFSSFNRFTTIFVCVLLCVVYLLSYASGEYDGRDSSSGSNNDRAPSNEFGSCTDGKCIKRTSQDI  TSGMWFGPRLGRRRRADRKPEINSDIEAFANAFEEPHWAIVTIPETEKRQITQFTPRLGRESGEDYFSYG  FPKDQEELYTEEQIYLPLFASRLGRRVPWTPSPRLGRQLHNIVDKPRQNFNDPRF |
| >gi\|571538111\|ref\|XP_006559865.1\| PREDICTED: uncharacterized protein LOC100577997 isoform X2 [*Apis mellifera*]  MRNHLFVFLVVLSIFSVSLNLSPCEQDRQLESTRGGEKLKPNMRRAFGLLTYPRIGRSNAPISNLNFNRR  GVESDTDFQFYSAELDPAPDKDYEDSPAPKSLGRSMHAKHADRIPKEASWLISDRPRSSKDGSWKIDEGR  SIYPFLLNSDSRNSQVNGYTPTRLDRRGNDADRILRK |
| >gi\|282598181\|gb\|ADA83379.1\| PBAN-type neuropeptide precursor [*Rhodnius prolixus*]  MVSVSLVGLLLVALQLITNGCTQGTRRTLSKWHGDNDLTRQEETVMELLKDNPWAHFSVREGGRNTVNFSPRLGRDEEVVFTETSRSPPFAPRLGRIVFRPRFGRLTLAAQH |
| >gi\|151564696\|gb\|ABS17680.1\| CAP2b/CAPA prepropeptide [*Rhodnius prolixus*]  MSYTVGTILVTAVLVSTCAVINSAEQTNEDKTNTTLRIKRSPISSVGLFPFLRAGRARNFPATWGMLVGDDKNKREGGFISFPRVGRSGPKRNGGGGNGGGLWFGPRLGRNQKRGDSWTLEQLQPNLLPGYPAYNEEEKENQFSEELSDESVSNKIV |
| >gi\|732592999\|gb\|JAG11923.1\| hypothetical protein CM83_16454 [Lygus hesperus]  MVNLTATLQAALFVVLLLTVIHGMEEGGRDMRSVGNNFGGGSWSQDAHTNGNEFNGDEAIADLLKVSPWTFYANQGKLTFARESRNSASFQPRSGRDEESQFTETSRSPPFAPRLGRVLHYSPRFRRITLN |
| >gi\|732618189\|gb\|JAG24518.1\| Cardio acceleratory peptide 2b, partial [*Lygus hesperus*]  CAVATKINHPIMIALGSLVLAFLTYHHLVVESQAAVTGRMNRAEAQRVNRDTSGLIPFPRVGRARIALSPLSFLPSLNFDNAQMLRLASPVESDETSWDLITDILKKQESGLIPFPRSGRSGTGPKRNGAGSGGSLWFGPRLGKRTYNLNNIGSADTNALTSVPDFFNQNKMAYDDSAASVKMGELQ |
| >gi\|17737929\|ref\|NP_524329.1\| hugin [*Drosophila melanogaster*]  MCGPSYCTLLLIAASCYILVCSHAKSLQGTSKLDLGNHISAGSARGSLSPASPALSEARQKRAMGDYKELTDIIDELEENSLAQKASATMQVAAMPPQGQEFDLDTMPPLTYYLLLQKLRQLQSNGEPAYRVRTPRLGRSIDSWRLLDAEGATGMAGGEEAIGGQFMQRMVKKSVPFKPRLGKRAQVCGGD |
| >gi\|23172626\|gb\|AAF56969.2\| capability [*Drosophila melanogaster*]  MKSMLVHIVLVIFIIAEFSTAETDHDKNRRGANMGLYAFPRVGRSDPSLANSLRDGLEAGVLDGIYGDASQEDYNEADFQKKASGLVAFPRVGRGDAELRKWAHLLALQQVLDKRTGPSASSGLWFGPRLGKRSVDAKSFADISKGQKELN |
| >gi\|108871571\|gb\|EAT35796.1\| AAEL012060-PA [*Aedes aegypti*]  MFRLYFFFNVICIFLAIRSAIGGEVPDATEQKINNFLASGKDSEDLSKRAAAMWFGPRLGKRTIASELHDEMMDEIDDNPLYYSGESPQRVASEIAQGTPYVVLLLTGRVLRQPQPVFYHSTTPRLGRRDASSSNENNSRPPFAPRLGRNLPFSPRLGRSFGAPVVDNFAY |
| >gi\|108878864\|gb\|EAT43089.1\| AAEL005444-PA [*Aedes aegypti*]  MSHRFNLSSDLDSVSEGRHKRGPTVGLFAFPRVGRSDPDLLEWSDAAAVAAALPLELADDYEDYPIREAKRQGLVPFPRVGRSGMNAARFYWPKTMMPQQQKRAGNSGANSGMWFGPRLGKRANAASTEIKGTEVYTPRLGRNSERPQIGESGDLNARSSSRSKLEDFERLFRSSDN |
| >gi\|58376073\|ref\|XP_307885.2\| AGAP002292-PA [*Anopheles gambiae* str. PEST]  MSRFYFFFNLICLYLAIKSALSAELDTNDQKYADLRTTGRGESPDSTGPDSDTLRRDDGAEGLNKRAAAMWFGPRLGKRTIAADLHDDLVEEFDAEPLGYAGEPPQKLATELVQGAPYMVLLVTAKPRKPQPIFYHTTSPRLGRRDSVGENHQRPPFAPRLGRNLPFSPRLGRSYNGGYPLPFQFAY |
| >gi\|119114897\|ref\|XP_566030.2\| AGAP000347-PA [*Anopheles gambiae* str. PEST]  MLAGSQAKPPVCVALALVLLGVTVHLAGAEAPEFESVGRVSKRGPTVGLFAFPRVGRSDPELNLDWESSAMLPLETADDYEDYPMKEMKRQGLVPFPRVGRSGKSELAMAAARYWQAARNLQQQQQQQSVVKRAGGTGANSAMWFGPRLGKRSRFGAAAASGSSEQQQQLKAEQL |
| >gi\|668459015\|gb\|KFB46855.1\| AGAP002292-PA-like protein [*Anopheles sinensis*]  MFRFYFFFNLICLYLAIKSALSVEMETNDQKFGHEMTDGATETDGLRREDGEDGINKRAAAMWFGPRLGKRTIPVDLHDELVEEFDSEPLGYAGESPEKLATDLIEGTPYVVVLLTARPRKPQPIFYHTTSPRLGRRDSVGENHQRPPFAPRLGRNLPFSPRLGRSYNGGYPLPLPFQLSY |
| >gi\|668450061\|gb\|KFB39390.1\| AGAP000347-PA-like protein [*Anopheles sinensis*]  MTLHIEFDVGSRSKRGPTVGLFAFPRVGRSDPELASVDWDPAGVLPAVDMADDYEDYPIKEIKRQGLVPFPRVGRSGVGAKSEVGGAAAARYWLVARALQQQQRALQQQMLGQPSVVKRAGGSGANSGMWFGPRLGKRSRSGTSSQAGSAEQQQQQQQQQLAKADQL |
| >gi\|646691513\|gb\|KDR07140.1\| PBAN-type neuropeptide [*Zootermopsis nevadensis*]  MRTDFSTQQHLIHTIVLLCLVVALASCDGFRLSSDPLEDGLLLGLEGLGDDPLAAKRGEPEVTGMWFGPRLGRREKRSVDDFPEDVADIRVEEVMELLKDTPWALLPLRGGKRHIEGFVPRLGRDSNEDEDADMMEQRSPPFAPRLGRRLVPFRPRMGRDRLPHDVYSPRLGRSVPHEKKQTPPHH |
| >gi\|646707069\|gb\|KDR13989.1\| hypothetical protein L798_12162 [*Zootermopsis nevadensis*]  MKQILFSCAMIHVLLLVSSVQCDEESDSVSTNTDAERGSSGLIPMPRVGRSDLAWTLQRQGDTVPSSLINRRSSSGLISMPRVGRGFLGLAPGVRTDPYLKDKRGSSGLIPMPRVGRSDVFWPLTDAFNVDGNKVGGLEKNINGDAGKTSTGMWFGPRLGKRREGNIDIPWAIVTVKEIPADVRDYTPYLTRESEGKEDYRVLLDEELPVRSGRIIHGHE |
| >gi\|242005997\|ref\|XP_002423845.1\| hypothetical protein Phum_PHUM088390 [*Pediculus humanus corporis*]  MEISIVKEILEKNVMWIGPRLGRRKRSDDDNTNQLDKNSGPPGVLELLQESPWAIVALKGPLRSSIGGKRRTVNFIPRLGRDSEEEYVDAPPDFAKSSSRSGNGISYGGGSNNFTPRLGRYVNLYGKDHL |
| >gi\|242005027\|ref\|XP_002423376.1\| conserved hypothetical protein [*Pediculus humanus corporis*]  MNIKSEARNPNVFICLMKMNPSVAFICCLCVVAVYSADEKNNKTKRDVSGLFPFPRVGRMDSSWNSRYLGPREMKRQG |
| >gi\|270015120\|gb\|EFA11568.1\| pyrokinin [*Tribolium castaneum*]  MERFILINWTVLCVAVLFFETVLSTPHESSVPNERNDDSKETYFWFGPRLGRKKRNSSNDDLYQDMQKEELVSLTDALQDVPWAIIAVNEGKRHVVNFTPRLGRESGEEFVNNAPEDRWLQNHETSGEMLYQRSPPFAPRLGRHSSPFSPRLGRENDRNLFS |
| >gi\|642921341\|ref\|XP_008192828.1\| PREDICTED: uncharacterized protein LOC103312856 isoform X1 [*Tribolium castaneum*]  MKTFLIYSACVVLFCIANCQGEPKEPKRNKLASVYALTPSLRVGRRSEGTDVKRRIGKMVSFPRIGRSESNWVPDDNSYGAQRPGANSGGMWFGPRLGRVQKRSENFTPWAYIILNGEAPIIREVHYSPRLGRESEEAYEEILDSNLDVL |
| >gi\|817067595\|ref\|XP_012255697.1\| PREDICTED: PBAN-type neuropeptides-like [*Athalia rosae*]  MTEPAAAHKRCNSQSKLEITEDKKIFIEWKLRNDTRESELNGVRMVEEGNICSGTSCAKRSSMNGVSGSMWFGPRLGRRRRSDEKLDIDPDETNAIADVINGAPWALVSIPAGKRHALQVFTPRLGRELGEDFGYSSLTGGQGGSQDDAEQRSPPFAPRLGRRLPFIPSPRLGRELRALFQKI |
| >gi\|817084715\|ref\|XP_012265014.1\| PREDICTED: uncharacterized protein LOC105691247 isoform X1 [*Athalia rosae*]  MKDTLSFVLVLLIATTSLNRGEKIKLPNNNKRYVGFLNVPRNGRSPGMTGYARTDRASGLMPFPRIGRSDPSDYDVVLDLNYDDMSHEVKRQGLIPFPRVGRSRDVSSSPYGNELVKVAKVKRTGDAVPNGGMWFGPRLGRLHRRADDPQPDDQQWAVFPLREFPAPGPQADNFTPRLGRESDGDETQSTPNTGGY |
| >gi\|357611058\|gb\|EHJ67284.1\| PBAN-type neuropeptide [*Danaus plexippus*]  MGCIWYFLLSFTILLFTLHLIGASDMKDDNLDRGAHSDRGGVWFGPRLGKRSLQLLDDSDSQTFVRLLEAAEALKYYYDQMSYQMQADAPQKVIKKVIFTPKLGRALDQYSERMAGNIDFTPRLGRKLPERTPTTSSDEDSIQDAIAANRRPSYFSPRLGRNYNFSPRLGRELYELYPEERVRVARSVNGTNSK |
| >gi\|357603706\|gb\|EHJ63890.1\| CAPA [*Danaus plexippus*]  MQSIAVVSLCLFFFTSVVSSSYHTSAKLRRDGVLNLYPFPRVGRASHTWQLPIKEKLPSPSVLDPESTKRQLYAFPRVGRDMPLEDLYHYIKRQSFEPQSKEVGMWFGPRLGRAYHGDDIEMTDNEAERGEQLEQNVSEREKRNAKEI |
| >gi\|27657760\|gb\|AAO18192.1\| pheromone biosynthesis activating neuropeptide PBAN [*Manduca sexta*]  MSQILIEFIALCLICVVTGSNDIKDEGDRGAHSDRGALWFGPRLGKRSLKLSSEDDRQAFFRLLESADTLKYYYDQLPYYERQIDEPAKVTKKVIFTPELGRSLDDSTQEKRVFYENFEFTPRLGRRISEDMPATPSDQEYPMYHPDPEQIDTRTRYFSPRLGRTHFSPRLGRELSYDMPTNVRVARSTNKTLN |
| >gi\|50080734\|gb\|AAT69684.1\| CAPA [*Manduca sexta*]  MQSAVRLVVCLFLLSSVLGGSYQSGPKLRRDGVLNLYPFPRVGRASHHTWQIPINDLYLEYDPVDKRQLYAFPRVGRSELSLLRPEQHLDALQPVPARRTEGPGMWFGPRLGRSFKSDEDEITIQNNNLERSEPELMERKKRNAHLN |
| >gi\|21205872\|gb\|AAM43840.1\|AF492474_1 diapause hormone and pheromone biosynthesis activating peptide [*Helicoverpa armigera*]  MFQTQLFVFLAVFTTSSVLGNNNDVKDGGAASGAHSDRLGLWFGPRLGKRSLRISTEDNRQAFFKLLEAADALKYYYDQLPYEMQADEPETRVTKKVIFTPKLGRSLAYDDKSFENVEFTPRLGRRLSDDMPATPADQEMYRQDPEQIDSRTKYFSPRLGRTMNFSPRLGRELSYDMIPNKIRVVRSANKTRST |
| >gi\|464897166\|gb\|AGH25549.1\| CAPA [*Helicoverpa armigera*]  MQPSMRIIVSMALLASALASAYHSSAKLRRDGVLNLYPFPRVGRASRNTWQLPLNDLYLEYEPSAEKRQLYAFPRVGRSDLSLSRPDSHEFQPMAVRRTESPGMWFGPRLGRSFKSDDDEIIIQNENTDHSEPEQTEPVHEDRRKRQTLN |
| >gi\|500862\|dbj\|BAA03755.1\| diapause hormone precursor [*Bombyx mori*]  MYKTNIVFNVLALALFSIFFASCTDMKDESDRGAHSERGALWFGPRLGKRSMKPSTEDNRQTFLRLLEAADALKFYYDQLPYERQADEPETKVTKKIIFTPKLGRSVANPRTHESLEFIPRLGRRLSEDMPATPADQEMYQPDPEEMESRTRYFSPRLGRTMSFSPRLGRELSYDYPTKYRVARSVNKTMDN |
| >gi\|195963369\|ref\|NP_001124357.1\| CAPA/CAP2b protein isoform A precursor [*Bombyx mori*]  MQSTMKLLTIITLISITFNSSHQSGAKLRPDGVLNLYPFPRVGRASYRTWQIPINDVYLDYEPVEKRQLYAFPRVGRGGPPSDRNEPHDDLLGLHLDDPGMWFGPRLGRSLKNGDDDVVNQNEDGRSEREQIDQIAHEERMKRRSKLL |
| >gi\|328715529\|ref\|XP_003245653.1\| PREDICTED: serine/threonine-protein kinase STE20-like [*Acyrthosiphon pisum*]  MCTVCYLWFILACSLLMTNANILNDLQEAQKFMEQLDYDYSAVVADAAAAAAQQRPVADLLWYDYGGGSGISGGVGRGESGGGSSGAGYFGGGFPVTGVQATPLFGADKRGGTTQSSNGGIWFGPRLGRRKRRGGSPFSGSVVHPVDGNAIGPAASSLLQQNPLAAGSTSAAAEQAAVSDLINNVPWVLVPIIDNSLYNQIQMKQNARNGRSSEEDDDDVASRSRHSARSPPYSPPFSPRLGRQAVMAQPQVPRLGRETLLYRRDARNALYQQSNATLLKQQRQQQQQQQQHSPQTAAESAAARRQAV |
| >gi\|240848823\|ref\|NP_001155835.1\| capability precursor [*Acyrthosiphon pisum*]  MKNLQTQIAAALLLTLTFFFTHALRHDSEYSDEYKRDNSRDRRESAVAGLIPFPRVGRSGINSALQMDNLYETQRELRSHKREGLIPFPRIGRRSESKNTALWFGPRLGRSVVIPESYDTSYLDSDTPTIIKTLIEMKSKNFGDEDDSLM |
| >gi\|701838142\|gb\|GBHB01029223.1\| TSA: *Teleogryllus commodus* MblContig29224 transcribed RNA sequence  AAGRPSSRAPAPPSKRAAGEDPVDAPSRTPRVDATVDAEDTDDAAGDGDTQLPAPSRADVLLLLDRDAEKSPVKRDGASAAGGMWFGPRMGRATDDQNVSPGMWFGPRMGRAAGGEDRTQASSGMWFGPRMGRATSAEEQPFSPGMWFGPRMGRSTATDEQNRQVATGMWFGPRMGRSGRTTAGAGDAARREHDLSSGMWFGPRMGRSAAASGVAQGGRRGADDVLLVFRRRQPADQAPGSGAQRPQELASGMWFGPRMGRRRDADWAGKRGPDSPEGMWFGPRLG |
| >gi\|639535833\|gb\|AUST01000043.1\| *Zootermopsis nevadensis* contig43, whole genome shotgun sequence  MNTQLLCSQHILIWFLVSAAVNNLCSVNTAMEETRIPGSDYGTVDGQRPSCKNKKSPAADVINLSVEFNMDHKNEKPNGTAESQPHIKLEDKIMNNFILENWSIPAEIIKNKTTTSLFTGQGDVETLMLQPTSNRMWYAPRLGRRDKRSRVNDEQVKQESARGGMWFGPRLGRRDKKSSINDEQVKQEFAYGGMWFGPRLGRRDKKSNVNDEQVKQEVAYGGMWFGPRLGRRDKKSNVHDEQVKQEVAHGGMWFGPRLGRREKKSYENEWPKKPRPFWMFLETTNKPREYDFLPQFDQDTVSEIYATAVNNITSELENYSSQLSDDIGIMKYSPSSRVRFSSQLGHKQKKATNVIAYVPQEVQMWTSEDKNNKPPEYDFTPRLGRELYEKGLTLASKSLRLPQIGHNTGIKYVYFQQD |
| >gi\|692774743\|gb\|JPZV01214316.1\| *Blattella germanica* strain American Cyanamid = Orlando Normal ContigNC214316, whole genome shotgun sequence  MRNFSSLIIWILSISISQSIYAADGFYHDSATSKTRDQSYFEDLLALCPNCRSDNLSIRELFKTPPKYLTTKSERLNSRTSDDKDTKFQTSKEDDVTYSAQPENSEWLQTIMEQRHKRSTYREPLQKEHKKKYLIVSNTEVGHNQKFTIGTTNKSSTTSGLEFSSKLGRQGKRSINSGEIERQSNQGEDRSINGEVMQYGPRDKKFGFWFQPKQFGIWFGPRLGKRDTKAAQMQENAGIWFGPRMGKRGKKTAQFQESAGKWFGPRMGKRDKRSAEMQQTAGIWFGPRMGKREEQSAQMQENAGIWFGPRMGKRDKKSVEMQESAGMWFGPRMGKRDKKSAEMRESAGMWFGPRMGKREEQSAQMQESAGIWFGPRMGKREDQSAQMQQSAGIWFGPRMGKRDKKSVEMQESAGMWFGPRMGKREDQSAQMQESTRIWFGPRMGKKDKKSAEMQESAGIWFGPRMGKRDKKSVEMQESAGIWFGPRMGKRDNKLAQFQESDGMWFGPRMGKREDQSAQIQESAGIWFGPRMGKRDKKPAKMQESAGMWFGPRMGKREDQSAQMQESAGIWFGPRMGKRDDKSAQMLENAGMWFGPRMGKREDQSAQMQESAGIWFGPRMGKRDDKSAQMLENVGMWFGPRMGKREEQSAQMQESAGMWFGPRMGKRDEKLVEDQVGAAEIWFGPRIGGREKKLEVKGGDVWNEPSITHTTMDICNRLRILGKENTENRCIEKKLFSDTAPMLWTLLSSTNSSSINYNFNPRLGRNIIGEDYPELSDDTYHTSNQLSDINAEELNERLIHMNNKLPHVSSLWSLVSNDNYLQNDVIPVSVDELYKNEHLSQRSTPTKSRNDIKYTYFTPRLGRTVT |
