# Supplementary material for: Novel Lom-dh Genes Play Potential Role in Promoting Egg Diapause of Locusta migratoria L
Source: Front Physiol. 2019 Jun 18;10:767. doi: 10.3389/fphys.2019.00767 (PMC6591537; doi:10.3389/fphys.2019.00767)
Supplement: TABLE S3 — Specific primers of this chapter. [file Table_3.DOCX]

**Table S3 Specific primers**

| Primer | Sequence（5'to3'） | Function |
| --- | --- | --- |
| Lom-dh1 F | ATGCCGAGAGGTGCTCAGCTATTC | Primer for cloning Lom-dh1 |
| Lom-dh1 R | TAGCACTGTTGTTTAACGGCCGAAG |  |
| Lom-dh4/5 F | ATGGAGAGCAGCATGGACGT | Primer for cloning Lom-dh4 and Lom-dh5 |
| Lom-dh4/5 R | TCAAAACCACATCCCTGGTTCTG |  |
| Lom-dh2/3 F | ATGCTGAGAAGAACACATTCGTCGC | Primer for cloning Lom-dh2 and Lom-dh3 |
| Lom-dh2/3 R | CTAGCGCAACTGCTCGCTTGC |  |
| Lom-capa F | ATGGCCGCCCCCAGCA | Primer for cloning Lom-capa |
| Lom-capa R | CTAGCGCTCCTCGGTGTC |  |
| Lom-pban F | ATGGCTCCAGCCGTGGC | Primer for cloning Lom-pban |
| Lom-pban R | CTACTCGTCGGCCGGCT |  |
| DNAcheck F | TGGAGACCTTCGCCTTTCA | Primer for detecting DNA pollution in cDNA |
| DNAcheck R | TCAGCGTCCTCTCACTCTTG |  |
| dsLom-dh1 F | TAATACGACTCACTATAGGATGCCGAGAGGTGCTCAGCTATTC | Primer for making dsLom-dh1 |
| dsLom-dh1 R | TAATACGACTCACTATAGGTAGCACTGTTGTTTAACGGCCGAAG |  |
| dsLom-dh4/5 F | TAATACGACTCACTATAGGATGGAGAGCAGCATGGACGT | Primer for making dsLom-dh4/5 |
| dsLom-dh4/5 R | TAATACGACTCACTATAGGTCAAAACCACATCCCTGGTTCTG |  |
| dsLom-dh2/3 F | TAATACGACTCACTATAGGAGAAGAACACATTCGTCGCTTG | Primer for making dsLom-dh2/3 |
| dsLom-dh2/3 R | TAATACGACTCACTATAGGGCGTGCATACTGTCCAAATGAC |  |
| dsGFP F | TAATACGACTCACTATAGGTACGACTCACTATAGGAGTAAAGG | Primer for making dsGFP |
| dsGFP R | TAATACGACTCACTATAGGTAGGTTTGTATAGTTCATCCATACC |  |
| rtActin F | GTTACAAACTGGGACGACAT | RT-PCR primer for reference gene |
| rtActin R | AGAAAGCACAGCCTGAATAG |  |
| rtLom-dh1 F | AATGTGGTGGTTCAGCGATG | RT-PCR primer for Lom-dh1 |
| rtLom-dh1 R | CTTTCTGTTGTCGTGCGGTG |  |
| rtLom-dh4/5 F | CGACAGGAACGAAGAAAGCA | RT-PCR primer for Lom-dh4/5 |
| rtLom-dh4/5 R | GAGGTCCAAACCACAAGCCT |  |
| rtLom-dh2/3 F | CAGTTCTTTCACGCAGCAAA | RT-PCR primer for Lom-dh2/3 |
| rtLom-dh2/3 R | CTTCAGGTTCGGAGTCACTATGA |  |
